# ANO1‐Mediated Inhibition of Cancer Ferroptosis Confers Immunotherapeutic Resistance through Recruiting Cancer‐Associated Fibroblasts

**DOI:** 10.1002/advs.202300881

**Published:** 2023-06-21

**Authors:** Fangli Jiang, Keren Jia, Yang Chen, Congcong Ji, Xiaoyi Chong, Zhongwu Li, Feilong Zhao, Yuezong Bai, Sai Ge, Jing Gao, Xiaotian Zhang, Jian Li, Lin Shen, Cheng Zhang

**Affiliations:** ^1^ Department of Gastrointestinal Oncology Key Laboratory of Carcinogenesis and Translational Research (Ministry of Education/Beijing) Peking University Cancer Hospital & Institute Beijing 100142 P. R. China; ^2^ Department of Pathology Key Laboratory of Carcinogenesis and Translational Research (Ministry of Education/Beijing) Peking University Cancer Hospital & Institute Beijing 100142 P. R. China; ^3^ Department of Medical Affairs 3D Medicines, Inc. Shanghai 201199 P. R. China; ^4^ Department of Oncology Shenzhen Key Laboratory of Gastrointestinal Cancer Translational Research Cancer Institute Peking University Shenzhen Hospital Shenzhen‐Peking University‐Hong Kong University of Science and Technology Medical Center Shenzhen 518000 P. R. China

**Keywords:** ANO1, cancer‐associated fibroblasts, ferroptosis, gastrointestinal cancers, immunotherapeutic resistance

## Abstract

The application of immunotherapy in gastrointestinal (GI) cancers remains challenging because of the limited response rate and emerging therapeutic resistance. Combining clinical cohorts, multi‐omics study, and functional/molecular experiments, it is found that ANO1 amplification or high‐expression predicts poor outcomes and resistance to immunotherapy for GI cancer patients. Knocking‐down or inhibiting ANO1 suppresses the growth/metastasis/invasion of multiple GI cancer cell lines, cell‐derived xenograft, and patient‐derived xenograft models. ANO1 contributes to an immune‐suppressive tumor microenvironment and induces acquired resistance to anti‐PD‐1 immunotherapy, while ANO1 knockdown or inhibition enhances immunotherapeutic effectiveness and overcomes resistance to immunotherapy. Mechanistically, through inhibiting cancer ferroptosis in a PI3K‐Akt signaling‐dependent manner, ANO1 enhances tumor progression and facilitates cancer‐associated fibroblast recruitment by promoting TGF‐*β* release, thus crippling CD8^+^ T cell‐mediated anti‐tumor immunity and generating resistance to immunotherapy. This work highlights ANO1's role in mediating tumor immune microenvironment remodeling and immunotherapeutic resistance, and introduces ANO1 as a promising target for GI cancers’ precision treatment.

## Graphical abstract

This work highlights ANO1 as a promising target for gastrointestinal (GI) cancers’ precision treatment. ANO1 is highly amplified or expressed in GI cancers. Through inhibiting cancer cell ferroptosis in a PI3K‐Akt‐dependent manner, ANO1 stimulates the production and secretion of TGF‐*β* by cancer cells, subsequently strengthens cancer‐associated fibroblast recruitment, and cripples CD8^+^ T cell‐mediated anti‐tumor immune responses, generating resistance to immunotherapy.

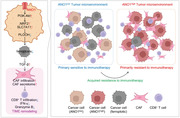

## 1 Introduction

Gastrointestinal (GI) cancers, majorly including gastric cancer (GC), esophageal cancer (EC), and colorectal cancer (CRC), represents a main cause of cancer death around the world.^[^ ^1^, ^2^ ^]^ Originated from the digestive tract, GI cancers shared diverse histopathological traits and molecular backgrounds, yet possess similarities such as quiescent tumor immune microenvironment (TIME) and lack of therapeutic biomarkers and targets. The rapid development of immunotherapy has greatly boosted GI cancers’ treatment.^[^ ^3^, ^4^ ^]^ The combination of chemotherapy with anti‐PD‐1 antibodies, representatively Pembrolizumab and Nivolumab, has been approved as the standardized regimen against GI cancers,^[^ ^5^, ^6^ ^]^ bringing hope for “homotherapy for heteropathy.” However, because of their complicated tissue composition and mutational heterogeneity, advanced GI cancers achieve limited therapeutic benefits from existing immunotherapy strategies.^[^ ^7^ ^]^ Certain genomic and molecular features, specifically microsatellite instability (MSI)/deficient mismatch repair (dMMR) and high PD‐L1 expression (represented by combined positive score, CPS), were currently considered as profitable biomarkers for immunotherapy, yet these features are still imperfect as predictors for immunotherapeutic outcomes since they cover only a small part of GI cancer population. On the other hand, primary/acquired resistance remains to be a principal factor that limiting immunotherapy from achieving cure.^[^ ^8^, ^9^ ^]^ Therefore, it is urgently needed to explore novel biomarkers and targets to improve the efficacy of immunotherapy for GI cancers.

As a calcium‐activated chloride channel protein, ANO1 (TMEM16A) plays important physiological functions such as transepithelial ion transport, smooth muscle contraction, and glandular secretion.^[^ ^10^, ^11^ ^]^ ANO1 has been reported to be a valuable target in noncancerous diseases. Cystic fibrosis, a hereditary disease induced by the dysfunction of cystic fibrosis transmembrane conductance regulator chloride channel, could be benefited from activating ANO1 as an alternative Cl^−^ channel.^[^ ^12^, ^13^ ^]^ Ca^2+^‐activated chloride currents in vascular smooth muscle cells are candidates for increasing vascular contractility, outlining ANO1 as a promising target for the treatment of hypertension.^[^ ^14^ ^]^ In cancer, ANO1 was originally recognized to be positively expressed in gastrointestinal stromal tumor (GIST) and was more well‐recognized as DOG1 (Discovered On GISTs protein 1).^[^ ^15^ ^]^ ANO1 was overexpressed at both mRNA and protein levels in esophageal squamous cell carcinoma, while its expression was positively correlated with lymph node metastasis and advanced clinical stage.^[^ ^16^ ^]^ ANO1 was also identified highly expressed in GC and CRC tissues, and was found to promote the growth of metastatic GI cancer cells.^[^ ^17^, ^18^ ^]^ Apart from GI cancers, ANO1 has also been noticed harboring high expression level in breast and ovarian cancer, and was associated with several malignant phenotypes.^[^ ^19^, ^20^, ^21^ ^]^ However, despite these preliminary reports, the exact role and concrete mechanisms of ANO1 in GI cancer progression and TIME regulation remain largely unelucidated, while its linkage with immunotherapy has never been explored. On the other hand, although several inhibitors targeting ANO1 has been developed,^[^ ^13^, ^22^ ^]^ their potential effectiveness against cancer remain unexplored yet by clinical trials because of the lack of strong preclinical evidence supporting ANO1's value as a promising therapeutic target in cancer.

Ferroptosis is a programmed cell death driven by accumulation of iron‐dependent lipid peroxides (lipid‐ROS), and plays vital role in modulating tumor progression and immune responses. Its repression is a frequent event in tumor and immune suppressive cells, while its induction could strengthen anti‐tumor immunity.^[^ ^23^, ^24^, ^25^ ^]^ On the other hand, cancer‐associated fibroblasts (CAFs) are the major type of stromal cells that robustly cross‐talk with TIME through secreting chemokines,^[^ ^26^ ^]^ thus supporting cancer nesting/proliferation/invasiveness/immune escape and therapeutic resistance.^[^ ^27^, ^28^ ^]^ Eliminating CAFs from the TIME is expected to be an effective anti‐cancer strategy, and combining immunotherapies and CAF‐targeted therapies has been under investigation by many clinical trials.^[^ ^29^ ^]^

In this study, through performing comprehensive investigations with GI cancer patient cohorts, bioinformatics datasets, and in vitro/in vivo models, we discovered ANO1 suppresses cancer ferroptosis in a PI3K‐Akt signaling‐dependent manner, thus facilitating CAF recruitment through promoting TGF‐*β* release, subsequently compromising CD8^+^ T cell‐mediated anti‐tumor immunity and inducing resistance to immunotherapy. In addition to revealing ANO1's role in TIME modeling, our work also provided solid preclinical evidences to support ANO1's potential as a therapeutic target for GI cancers’ precision treatment.

## 2 Results

### 2.1 ANO1 Amplification or High‐Expression Predicts Adverse Immunotherapeutic Outcomes

**Figure 1:**
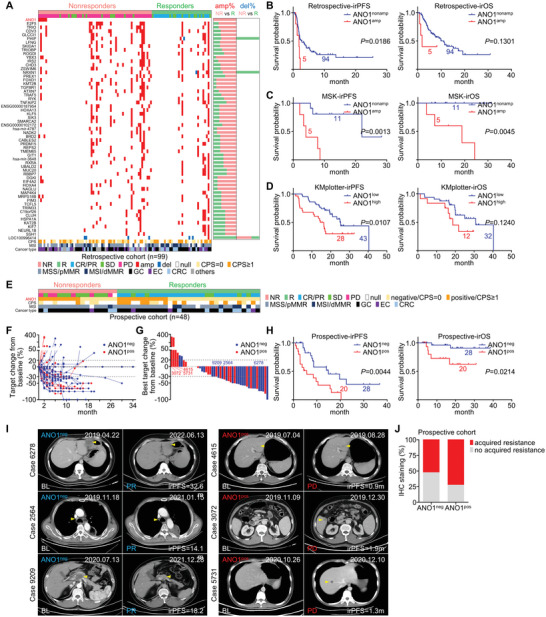
ANO1 amplification or high‐expression predicts adverse immunotherapeutic outcomes. A) A landscape of CNAs with >5% incident rate in a GI cancer retrospective training cohort received immunotherapy. The irPFS/irOS were stratified by ANO1 amplification for B) training cohort and C) MSK datasets, or stratified by ANO1 expression for D) KMplotter dataset of EC. E) The mutual relationship of response, IHC‐based ANO1 expression, CPS status, MSI status, and cancer types for a GI cancer prospective validating cohort received immunotherapy. F) The dynamic fold changes of target tumor volume from immunotherapy baseline to the end of follow‐up were measured for each patient of the validating cohort. G) The best fold changes of target tumor volume from the maximum tumor reduction to immunotherapy baseline were measured for each patient of the validating cohort. H) The irPFS/irOS for validating cohort were stratified by ANO1 positivity. I) Representative CT images during immunotherapy for patients of validating cohort with characterized ANO1 positivity at baseline. The target tumor measured was marked with arrowheads. J) Comparison of acquired immunotherapeutic resistance between ANO1‐negative and ‐positive responders in the prospective cohort. NR, nonresponder. R, responder. amp, amplification. del, deletion. neg, negative. pos, positive.

We referred to a previous study of our group, in which comprehensive genomic profiling was performed for the baseline tissue of 99 retrospective GI cancer patients treated with immunotherapy agents^[^ ^30^ ^]^ (training cohort, Table S1 and Figure S1A, Supporting Information) to determine their copy‐number alterations (CNAs). Among all candidate CNAs with >5% frequency, ANO1 was solely observed amplified in nonresponders (7.7%, 5/65) instead of responders (0%, 0/34) (**Figure** **1**A), while ANO1‐amplified patients displayed worse immunotherapy‐related progression‐free survival (irPFS, with statistical significance) and overall survival (irOS, with statistical tendency) than ANO1‐nonamplified patients (Figure 1B). ANO1 amplification's predictive value to adverse immunotherapeutic outcome retained for either GC (with statistical significance) or nonGC (EC+CRC+other cancers, with statistical tendency) (Figure S1B, Supporting Information). We also referred to other immunotherapy‐related datasets. In the genomic profiling‐based GI cancer MSK dataset (Figure S1C, Supporting Information), ANO1 amplified in 31.25% (5/16) patients and was drastically correlated with poor irPFS/irOS (Figure 1C), whose indication to adverse immunotherapeutic outcome retained for GC (Figure S1D, Supporting Information). According to the public dataset from the Kaplan–Meier Plotter (http://kmplot.com), ANO1 expression predicted unfavorable irPFS and irOS for EC (Figure 1D). In the RNA sequencing‐based melanoma GSE78220 dataset, ANO1 expression was evidently higher in nonresponders than in responders while also predicted unfavorable trend of irOS (Figure S1E,F, Supporting Information). It also should be noted that GIST, a tumor generally bearing ANO1 positivity, was known for poor immunotherapeutic responses.^[^ ^31^ ^]^ Among four GIST patients treated with immunotherapy in our center during 2018–2022 (Figure S1G, Supporting Information), immunohistochemistry (IHC) indicated that ANO1‐negative cases displayed potentially better irPFS/irOS than ANO1‐positive cases (Figure S1H, Supporting Information). Thus, despite insignificant statistics caused by limited sample size, ANO1 amplification/upregulation retrospectively indicated adverse immunotherapeutic outcomes in multiple cancer types.

In order to validate ANO1's value in directing immunotherapy, we recruited 48 GI cancer patients as the prospective cohort (Figure 1E) from two observational clinical trials (NCT04993378/NCT05427227) registered by our group (Table S2 and Figure S1I, Supporting Information), and evaluated ANO1 status with IHC before administrating immunotherapy (Figure S1J, Supporting Information). ANO1's positive rate was higher in nonresponders (52.9%, 9/17) than responders (35.5%, 11/31) (Figure 1E), while ANO1‐negative cases displayed more favorable dynamic changes of tumor volume (Figure 1F) and the best changes of tumor volume (Figure 1G) than ANO1‐positive cases. ANO1‐negative cases possessed longer irPFS/irOS than ANO1‐positive cases (Figure 1H), which retained either in GC (with statistical significance/tendency for irPFS/irOS) or nonGC (EC+CRC+other cancers, with statistical tendency) (Figure S1K, Supporting Information). Computer tomography (CT)‐recorded changes of tumor volume for representative cases were displayed (Figure 1I). Furthermore, among all responders, ANO1‐positive cases displayed higher rate of acquired immunotherapeutic resistance than ANO1‐negative cases (Figure 1J). It should also be noted that in both retrospective and prospective cohorts, ANO1 was largely irrelevant with major clinicopathological parameters (Tables S1 and S2 and Figure S1J, Supporting Information) and also displayed adverse prognostic trend according to COX regression analysis (Tables S3 and S4, Supporting Information), emphasizing ANO1 is a novel predictor for adverse immunotherapeutic outcomes of GI cancers.

### 2.2 The Genetic and Expressional Landscape of ANO1 in GI Cancers

According to The Cancer Genome Atlas (TCGA) datasets, ANO1's major form of genetic aberrance was amplification, rather than mutations. ANO1 amplified in >35% EC (esophageal carcinoma, ESCA), >6% GC (stomach adenocarcinoma, STAD), and >5% cholangiocarcinoma, and also frequently amplified in other non‐GI cancers, including head and neck squamous cell carcinoma, lung squamous cell carcinoma, breast invasive carcinoma, and bladder urothelial carcinoma (Figure S2A, Supporting Information). Transcriptionally, ANO1's expression was higher in GC/EC/CRC tumor than in normal tissue (Figure S2B, Supporting Information), while ANO1‐amplified patients displayed higher ANO1 expression than nonamplified patients according to TCGA datasets (Figure S2C, Supporting Information). On protein level, immunohistochemistry staining of 70 surgically resected GC cases (representatively in Figure S2D, Supporting Information) indicated evidently higher ANO1's positive rate in tumor than in normal tissue (38.6% vs 12.5%, Figure S2E, Supporting Information), while similar result was observed in another 30 GC cases (33.3% vs 23.3%, Figure S2F, Supporting Information) from a tissue microarray. ANO1 also displayed high positive rate (61.1%) in EC according to IHC of 20 surgically resected cases (Figure S2G, Supporting Information). In accordance with tissue, ANO1 was prevalently expressed in human/mice GI cancer cell lines (Figure S2H, Supporting Information). Additionally, high ANO1 transcript level was correlated with unfavorable OS in multiple GC datasets (Figure S2I, Supporting Information). Among the most important CNAs/mutations/gene expressions/TIME traits serving as biomarkers and targets for GI cancers’ targeted therapy and immunotherapy, ANO1's transcript expression was only positively correlated with its own copy number and EGFR expression for both TCGA‐STAD and ‐ESCA datasets (Figure S2J, Supporting Information), suggesting ANO1's distribution in population is largely independent to GI cancers' crucial molecular features.

### 2.3 ANO1 Facilitates the Malignant Progression of GC/CRC

**Figure 2:**
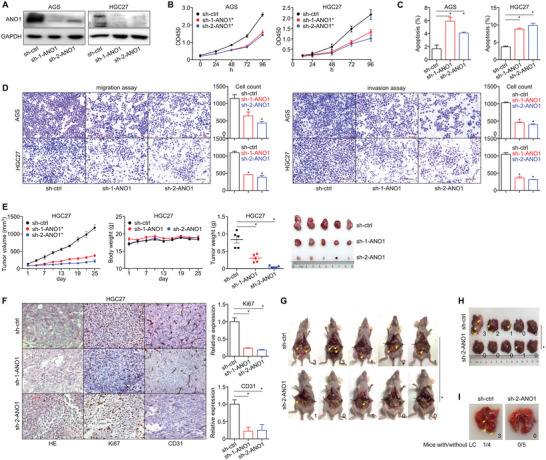
ANO1 knockdown inhibits the malignant progression of GC. A) ANO1 knockdown in AGS/HGC27 cells. Changes of in vitro B) proliferation, C) apoptosis, and D) invasiveness were measured after ANO1 knockdown. For GC CDXs, changes of their in vivo E) tumor growth, F) IHC‐based xenograft expression of Ki67/CD31, G) peritoneal metastasis, H) liver metastasis, and I) lung metastasis were measured after ANO1 knockdown. The number of metastatic lesions was marked for each model. *p < 0.05. Error bars, mean ± SEM.

We selected GC, the most representative type of GI cancers, to evaluate ANO1's malignant role. shRNA stably and significantly knocked‐down ANO1 expression for GC cell lines AGS/HGC27 and CRC cell line HCT116 (**Figure** **2**A and Figure S3A, Supporting Information). The in vitro proliferation rates were prominently dropped (Figure 2B and Figure S3B, Supporting Information) while the apoptosis rates were enhanced (Figure 2C and Figure S3C, Supporting Information), accompanied with attenuated migration/invasion capability after ANO1 knockdown (Figure 2D and Figure S3D, Supporting Information). These data suggested ANO1 facilitate GC's malignant progression in vitro. For cell‐derived xenograft (CDX) models, ANO1 knockdown leaded to markedly lower in vivo tumor growth rate/weight at the final observation (Figure 2E), and reduced IHC staining for proliferation marker Ki67 and vascular marker CD31 in stripped xenografts (Figure 2F).

To confirm ANO1's impact on in vivo metastasis, we constructed peritoneal colonization model through intraperitoneal injection, local liver metastasis model through spleen injection, and distant lung metastasis model through caudal vein injection. ANO1 knockdown consistently reduced the number of metastatic tumor nodules in the abdominal cavity (Figure 2G), liver (Figure 2H), and lung (Figure 2I), suggesting that ANO1 facilitates the growth and metastasis of GC.

### 2.4 ANO1 Is a Druggable Target for the Targeted Treatment of GI Cancers

**Figure 3:**
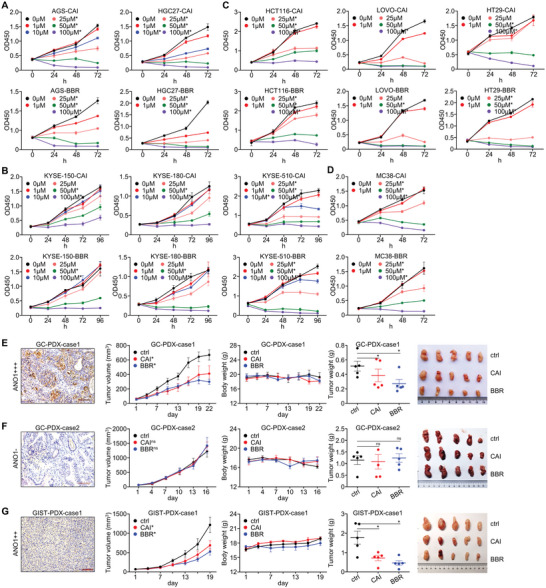
ANO1 is a druggable target for the targeted treatment of GI cancers. Inhibition effectiveness of CAI/BBR on the in vitro proliferation were measured with multiple A) human GC, B) EC, C) CRC cell lines, and D) mouse CRC cell line MC38. Inhibition effectiveness of CAI/BBR on the in vivo tumor growth were measure with E) an ANO1‐positive GC PDX, F) an ANO1‐negative GC PDX, and G) a GIST PDX with medium ANO1 expression. *p < 0.05. ns, not significant. Error bars, mean ± SEM.

In order to further validate ANO1's potential as a therapeutic target for GI cancers, we assessed CaCCinh‐A01 (CAI) and benzbromarone (BBR), two previously reported inhibitors targeting ANO1,^[^ ^12^, ^22^ ^]^ in cell lines and patient‐derived xenograft (PDX) models. CAI/BBR were feasible to inhibit ANO1's protein expression (Figure S4A,B, Supporting Information) and the in vitro proliferation of multiple human GC (**Figure** **3**A), EC (Figure 3B), CRC cell lines (Figure 3C) as well as a mouse cancer cell line MC38 (Figure 3D). Also, CAI/BBR selectively showed anti‐tumor effectiveness in an ANO1‐positive GC PDX (Figure 3E) rather than in an ANO1‐negative GC PDX (Figure 3F), and were both valid to inhibit the growth of a GIST PDX model with medium ANO1 positivity (Figure 3G). These findings supported that ANO1 is a druggable target for GI cancers’ targeted treatment.

### 2.5 ANO1 Contributes to an Immune‐Suppressive Tumor Microenvironment

**Figure 4:**
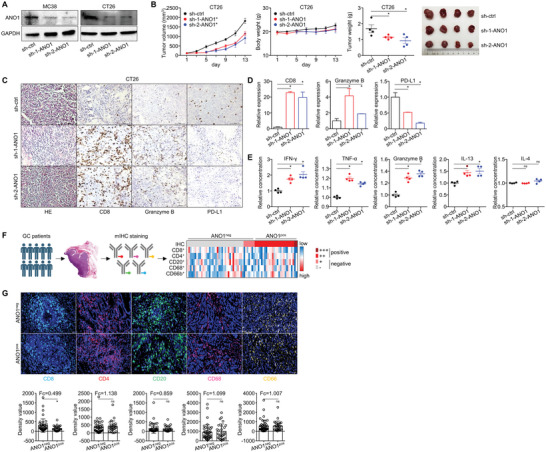
ANO1 contributes to an immune‐suppressive tumor microenvironment. A) ANO1 knockdown in MC38/CT26 cells. For CT26 CDX, changes of in vivo B) tumor growth, C) IHC‐staining of infiltrated CD8/granzyme B/PD‐L1, D) IHC‐based quantification of infiltrated CD8/granzyme B/PD‐L1, and E) ELISA‐based expression of IFN‐*γ*/TNF‐*α*/granzyme B/IL‐13/IL‐4 in xenograft tumors were measured after ANO1 knockdown. F) An overview for mIHC‐categorized density of five major immune cells in TIME of 70 GC patients. G) Representative images and statistics of each major immune cells in GC patient specimens, whose density were stratified by ANO1 positivity. *p < 0.05. ns, not significant. Error bars, mean ± SEM.

We have proved ANO1 promoted GC's malignant progression under both in vitro and immunodeficient in vivo conditions. Since ANO1‐amplification/upregulation predicted poor immunotherapeutic outcomes, its role in uncompromised TIME should be discussed. ANO1 knockdown was performed for mouse colon cancer (COAD) cell lines (**Figure** **4**A). Comparable to the results in immunodeficient human cancer CDXs, ANO1 knockdown reduced the growth of CT26 CDXs (Figure 4B), paired with increased CD8/granzyme B and decreased PD‐L1 IHC staining in xenograft tumors (Figure 4C,D), suggesting a higher infiltration of cytotoxic CD8^+^ T cells in TIME. According to ELISA for mice CDXs, the concentrations of anti‐tumor cytokines IFN‐*γ*/granzyme B/TNF‐*α*/IL‐13 in xenograft tumors significantly elevated, while immunosuppressive cytokine IL4 remained largely unchanged after ANO1 knockdown (Figure 4E). We also performed multiplex IHC (mIHC) staining of major immune cells for the previously mentioned 70 GC surgery cases (Figure 4F), in which CD8^+^ T cell's infiltration was significantly lower in ANO1‐positive group than ANO1‐negative group (Figure 4G). As a result, ANO1 contributes to an immune‐suppressive tumor microenvironment, while ANO1 knockdown can restore the immune activity in TIME.

### 2.6 ANO1 Inhibition Augments the Effectiveness of Anti‐PD‐1 Immunotherapy against GI Cancers

**Figure 5:**
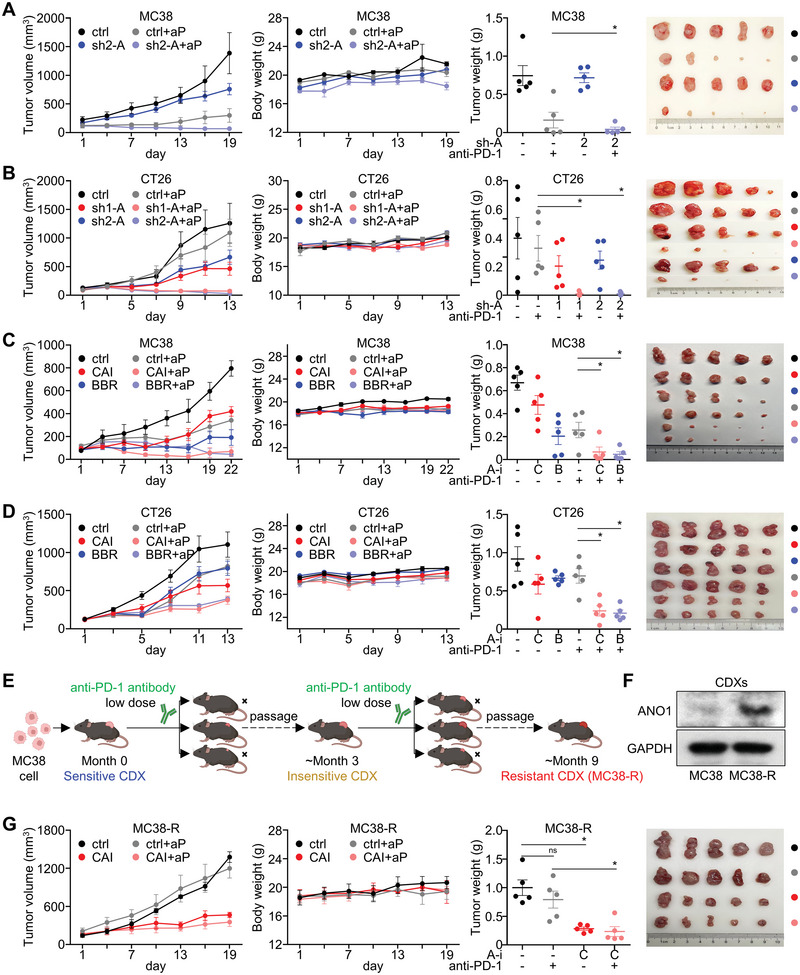
Inhibiting ANO1 augments the effectiveness of anti‐PD‐1 immunotherapy against GI cancers. The effectiveness of anti‐PD‐1 antibody (aP) in A) MC38‐derived xenografts and B) CT26‐derived xenografts after ANO1 knockdown. The effectiveness of anti‐PD‐1 antibody in C) MC38‐derived xenografts and D) CT26‐derived xenografts after treated with ANO1 inhibitors CAI/BBR. E) A flow chart for the establishment of a MC38‐R string with acquired resistance to anti‐PD‐1 antibody. F) Expression of ANO1 in MC38‐R CDX after acquired resistance to anti‐PD‐1 antibody. G) The effectiveness of anti‐PD‐1 antibody in MC38‐R CDXs after treated with CAI. *p < 0.05. ns, not significant. Error bars, mean ± SEM.

Since ANO1 knockdown could revoke the immune‐suppressive tumor microenvironment, we evaluated the impact of inhibiting ANO1 on the effectiveness of anti‐PD‐1 antibody in MC38‐ or CT26‐engrafted C57BL‐6J/BALB‐C mice models. In accordance with previous reports,^[^ ^32^ ^]^ MC38 xenograft displayed high sensitivity (**Figure** **5**A), while CT26 xenograft displayed low sensitivity to anti‐PD‐1 antibody (Figure 5B). Both ANO1 knockdown (Figure 5A,B) and inhibitors (Figure 5C,D) evidently augmented the anti‐tumor effect of anti‐PD‐1 antibody against MC38/CT26 xenografts. We also established MC38‐R, a subline acquired resistance to immunotherapy through performing long‐term, low‐dose induction with anti‐PD‐1 antibody on MC38 (Figure 5E). We observed elevated ANO1 expression in MC38‐R CDX compared with its progenitor MC38 CDX (Figure 5F), emphasizing ANO1's involvement in the acquisition of resistance to anti‐PD‐1 immunotherapy. Of note, ANO1 inhibitor CAI also re‐sensitized anti‐PD‐1 antibody for MC38‐R CDX (Figure 5G). These evidences were in accordance with our findings that GI cancer patients harboring ANO1 amplification/upregulation displayed adverse immunotherapeutic outcomes. According to these results, inhibiting ANO1 indisputably augments immunotherapeutic effectiveness against GI cancers, and reversed the primary/acquired resistance to anti‐PD‐1 antibody.

### 2.7 ANO1 Promotes Cancer Progression and Impairs Immunotherapeutic Efficacy through Inhibiting Ferroptosis

**Figure 6:**
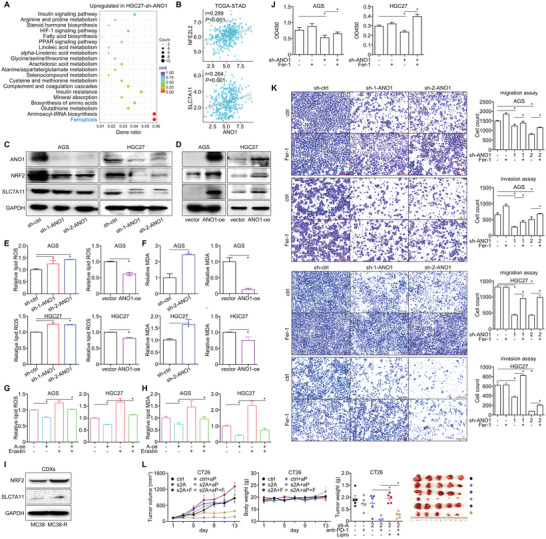
ANO1 promotes cancer progression and impairs immunotherapeutic efficacy through inhibiting ferroptosis. A) RNA‐sequencing identified genes upregulated in HGC27 cell after ANO1 knockdown were proceeded for KEGG enrichment analysis. B) Pearson correlation between expressions of ANO1 and NFE2L2/SLC7A11 in TCGA‐STAD dataset. Expression of ferroptotic proteins after C) ANO1 knockdown or D) overexpression (oe) were assessed. The levels of E) lipid ROS and F) MDA after ANO1 knockdown or overexpression were assessed. G) Lipid ROS and H) MDA repressed by ANO1 overexpression was rescued by ferroptosis agonist erastin. I) Expression of ferroptotic proteins in MC38‐R CDX after acquired resistance to anti‐PD‐1 antibody. The in vitro J) proliferation and K) invasiveness suppressed by ANO1 knockdown were reversed by ferroptosis inhibitor Fer‐1. L) The sensitized anti‐tumor effectiveness in CT26 CDX by combining ANO1 knockdown and anti‐PD1 antibodies was alleviated by ferroptosis inhibitor liproxstatin (Lipro). The color scale from blue to red reflected the *p* value from 1 to 0 (the smaller, the more significant). *p < 0.05. Error bars, mean ± SEM.

To unmask the mechanistic role of ANO1 in GI cancer, we carried out RNA‐sequencing for HGC27 cells before/after ANO1 knockdown. Referring to all the differentially expressed genes, ferroptosis was listed as the top enriched signaling (**Figure** **6**A). NRF2/SLC7A11, two crucial factors repressing ferroptosis, both displayed positive correlation with ANO1 in TCGA‐STAD dataset (Figure 6B) and were downregulated by ANO1 knockdown (Figure 6C and Figure S3E, Supporting Information) or upregulated by ANO1 overexpression (Figure 6D and Figure S3F, Supporting Information) on protein level in GC/CRC cells. Furthermore, lipid ROS (Figure 6E and Figure S3G, Supporting Information) and malondialdehyde (MDA) (Figure 6F and Figure S3H, Supporting Information), two main characteristics of ferroptosis, were substantially increased by ANO1 knockdown or reduced by ANO1 overexpression, while the inhibition of lipid ROS and MDA by ANO1 overexpression was reversed by the ferroptosis agonist erastin (Figure 6G,H and Figure S3I,J, Supporting Information). Furthermore, ferroptosis repressors NRF2/SLC7A11 were found upregulated after acquired resistance to immunotherapy (Figure 6I).

We then evaluated whether ANO1's malignant role relied on its inhibition on ferroptosis. The ferroptosis inhibitor Fer‐1 restored the inhibited in vitro proliferation (Figure 6J and Figure S3K, Supporting Information) and migration and invasion (Figure 6K and Figure S3L, Supporting Information) induced by ANO1 knockdown. In CT26 CDX, the sensitized anti‐tumor effectiveness by combining ANO1 knockdown and anti‐PD‐1 antibody was also alleviated by the ferroptosis inhibitor liproxstatin (Figure 6L). Taken together, ANO1 exerts its role in facilitating cancer progression and repressing immunotherapeutic responses through inhibiting cancer ferroptosis.

### 2.8 ANO1 Inhibits Ferroptosis through Activating PI3K‐Akt Signaling

**Figure 7:**
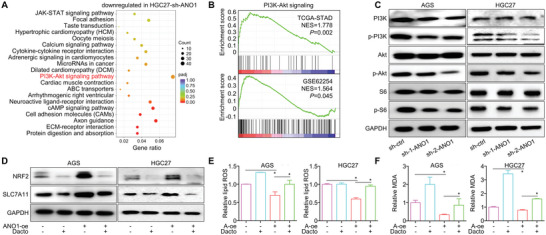
ANO1 impairs ferroptosis through activating PI3K‐Akt signaling. A) RNA‐sequencing identified genes downregulated in HGC27 cell after ANO1 knockdown were proceeded for KEGG enrichment analysis. B) ANO1‐related enrichment of PI3K‐Akt signaling in TCGA/GSE62254 datasets. C) ANO1 knockdown in GC cells deactivated PI3K‐Akt signaling. PI3K‐Akt signaling inhibitor Dactolisib (Dacto) abrogated D) the NRF2/SLC7A11 upregulation, and rescued the E) lipid ROS/F) MDA inhibition. The color scale from blue to red reflected the *p* value from 1 to 0 (the smaller, the more significant). *p < 0.05. Error bars, mean ± SEM.

We re‐analyzed the RNA‐sequencing for GC cells and found PI3K‐Akt signaling was enriched after ANO1 knockdown (**Figure** **7**A). In GC datasets TCGA/GSE62254, gene set enrichment analysis (GSEA) also suggested ANO1 was correlated with activated PI3K‐Akt signaling (geneset M271, Figure 7B). In GC/CRC cells, PI3K‐Akt signaling was inhibited by ANO1 knockdown (Figure 7C and Figure S3M, Supporting Information), marked by reduced phosphorylation of PI3K/Akt/S6. On the other hand, upregulation of ferroptosis decelerators NRF2/SLC7A11 (Figure 7D and Figure S3N, Supporting Information) and reduction of lipid ROS/MDA (Figure 7E,F and Figure S3O,P, Supporting Information) induced by overexpressing ANO1 were revoked by dactolisib, an PI3K‐Akt signaling inhibitor, suggesting that ANO1 inhibits ferroptosis through activating PI3K‐Akt signaling.

### 2.9 ANO1‐Mediated Ferroptosis Inhibition Provokes TGF‐*β* Production and Release and Recruits Cancer‐Associated Fibroblasts into TIME, Generating Resistance to Immunotherapy

**Figure 8:**
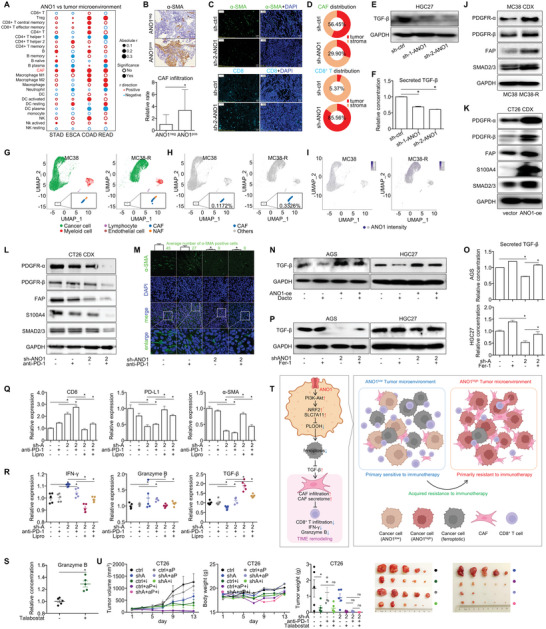
ANO1‐mediated ferroptosis inhibition provokes TGF‐*β* release and recruits cancer‐associated fibroblasts into TIME, generating resistance to immunotherapy. A) Correlation between ANO1 and all key microenvironmental cells in TCGA‐STAD/ESCA/COAD/READ datasets. B) The IHC‐based expression of infiltrated *α*‐SMA in prospective GI cancer patient‐cohort was stratified by the positivity of ANO1. C) The representative staining for CAFs and CD8^+^ T cells and D) their distribution ratio in tumor/stroma regions were assessed by mIHC analysis. The E) expression and F) secretion of TGF‐*β* by GC cell HGC27 were assessed after ANO1 knockdown. For MC38 and MC38‐R CDXs, the T‐distributed stochastic neighbor embedding (t‐SNE) maps of G) major cell components in TIME, H) CAF proportions, and I) intensity of ANO1 were assessed by single‐cell RNA sequencing. J) Expression of CAF markers in MC38/MC38‐R CDXs after acquired resistance to immunotherapy. K) Expression of CAF markers in CT26 CDX after ANO1 overexpression. L) Expression and M) fluorescent staining of CAF markers in CT26 CDX after ANO1 knockdown and anti‐PD‐1 antibody treatment. N) PI3K‐Akt signaling inhibitor Dactolisib (Dacto) repressed the TGF‐*β* upregulation induced by ANO1 overexpression. The O) expression and P) secretion of TGF‐*β* by GC cells were reduced by ANO1 knockdown and rescued by ferroptosis inhibitor Fer‐1. For CT26 CDX, changes of Q) IHC‐based expression of infiltrated CD8/PD‐L1/*α*‐SMA in xenograft tissue, and R) ELISA‐based expression of IFN‐*γ*/granzyme B/TGF‐*β* in CDX tissues were measured after combining ANO1 knockdown, anti‐PD‐1 antibody and liproxstatin treatment. S) The effect of talabostat mesylate on inducing granzyme B expression in CT26 CDX tissues. T) A schematic model summarizing ANO1's mechanism in mediating TIME remodeling and immunotherapeutic resistance. U) The tumor growth inhibition for the triple combination of ANO1 knockdown, anti‐PD‐1 antibody, and CAF inhibitor talabostat mesylate (talabostat) treatment. STAD, stomach adenocarcinoma. ESCA, esophageal carcinoma. COAD, colon cancer. READ, rectum cancer. CAF, cancer‐associated fibroblast. NAF, normal tissue‐associated fibroblast. *p < 0.05. ns, not significant. Error bars, mean ± SEM.

As one of the most abundant stromal cells in TIME, CAFs are critically involved in cancer progression. We quantified the proportion of all key microenvironmental cell types in TGCA‐STAD/ESCA/COAD/rectum cancer (READ) datasets with TIMER resource (http://timer.comp‐genomics.org/), then analyzed their correlation with ANO1's expression value. Among all key microenvironmental cells, CAF was the only type that consistently correlated with ANO1 expression across TCGA datasets of all four major GI cancers (**Figure** **8**A). According to the IHC staining of CAF marker *α*‐SMA in the previously analyzed 48‐case prospective GI cancer cohort, the relative infiltration rate of CAFs was significantly higher in ANO1‐positive patients than ANO1‐negative patients (Figure 8B). According to mIHC analysis, ANO1 knockdown reduced CAF infiltration while enhanced CD8^+^ T cell infiltration in CT26‐derived CDX tissue (Figure 8C). By further analyzing cells’ spatial traits in CT26 CDX tissue, reduced tumor/stroma ratio of CAF distribution and increased tumor/stroma ratio of CD8^+^ T cell distribution were observed after ANO1 knockdown (Figure 8D), while similar contexts were found for the density and area of CAFs and CD8^+^ T cells (Figure S5A,B, Supporting Information), indicating that ANO1 knockdown prominently induced a retreat of CAFs from tumor to stroma and an accumulation of CD8^+^ T cells from stroma to tumor. Across all four TCGA GI cancers, ANO1 expression displayed a high correlation with CAF‐related secretome members that drive malignant TIME remodeling (Figure S5C, Supporting Information), including TGF‐*β*, both a key product and a major stimulator of CAFs. ANO1 correlated with activated TGF‐*β* signaling according to GSEA for all four TCGA‐GI cancer datasets (geneset M18762, Figure S5D, Supporting Information), while we also noticed in GC/CRC cells that ANO1 knockdown repressed TGF‐*β* expression and secretion (Figure 8E,F and Figure S3Q,R, Supporting Information). These data concomitantly indicated ANO1 recruits CAF through promoting TGF‐*β* secretion by cancer cells.

We then explored whether CAFs were involved in the ANO1‐mediated resistance to immunotherapy, and whether TGF‐*β* stimulated CAFs could be regulated by the ANO1‐PI3K‐Akt‐ferroptosis signaling axis. After acquired resistance to immunotherapy, higher proportion of infiltrated CAFs and ANO1 expression were observed by single‐cell RNA sequencing for MC38/MC38‐R CDX tissues (Figure 8G–I), along with elevated protein expression of CAF biomarkers (PDGFR‐*α*/*β*/FAP) and TGF‐*β* signaling member SMAD2/3 (Figure 8J), suggesting the acquired resistance to anti‐PD‐1 immunotherapy was induced by ANO1‐recruited CAFs. In CT26 CDX tissue, CAF biomarkers were also consistently elevated after ANO1 overexpression (Figure 8K). Both the expression (Figure 8L) and immunofluorescent staining (Figure 8M) of CAF markers were drastically reduced in CT26 CDX tissue after combining ANO1 knockdown and anti‐PD‐1 antibody. On GC/CRC cellular level, PI3K‐Akt pathway inhibitor Dactolisib suppressed the upregulation of TGF‐*β* induced by ANO1 overexpression (Figure 8N and Figure S3S, Supporting Information), while the repression of TGF‐*β* expression/secretion by ANO1 knockdown were rescued by ferroptosis inhibitor Fer‐1 (Figure 8O,P and Figure S3T,U, Supporting Information), indicating TGF‐*β* production was regulated by the ANO1‐PI3K‐Akt‐ferroptosis axis. In CT26 CDX models with/without immunotherapy treatment, ANO1 knockdown increased CD8 infiltration and decreased PD‐L1/*α*‐SMA distribution in tissues, while in vivo ferroptosis inhibitor liproxstatin reversed these effects (Figure 8Q and Figure S5E, Supporting Information). Similarly, the expression of TGF‐*β* and other CAF markers in CT26 xenograft tissue were inhibited by ANO1 while were rescued by liproxstatin (Figure S5F, Supporting Information). In CT26 CDXs, ANO1 knockdown stimulated IFN‐*γ*/granzyme B and repressed TGF‐*β*, while these effects were reversed by liproxstatin (Figure 8R). These phenotypes about TGF‐*β*, CAFs, and CD8^+^ T cell were in concert with our previously described anti‐tumor effectiveness by combining ANO1 knockdown, anti‐PD‐1 immunotherapy, and ferroptosis inhibition (Figure 6L). In addition, we found in CT26 CDX that the sensitized anti‐tumor effectiveness by combining ANO1 knockdown and anti‐PD‐1 antibody was alleviated by TGF‐*β* treatment (Figure S6A,B, Supporting Information), while the increased CD8 infiltration and decreased *α*‐SMA distribution in CDX tissues induced by ANO1 knockdown were also reversed by TGF‐*β* treatment (Figure S6C,D, Supporting Information). These in vitro and in vivo data proved that the impact of ANO1‐PI3K‐Akt‐ferroptosis axis on immunotherapy efficacy, CD8^+^ T cell infiltration, and CAF recruitment depend on the participation of TGF‐*β*.

We further discussed the impact of ANO1‐recruited CAFs on CD8^+^ T cells, the major performer of anti‐tumor immunity. Referring to TIMER resource, we found in TCGA‐STAD/ESCA/COAD/READ datasets that high content of CAF was a precondition for the relative low content of CD8^+^ T cell in ANO1 highly expressed groups (Figure S7A, Supporting Information). Additionally, an anti‐CAF compound talabostat mesylate that inhibit in vivo FAP expression (Figure S7B, Supporting Information) could increase granzyme B expression in CT26 CDX (Figure 8S), proving direct clue that CAFs represses the function of cytotoxic CD8^+^ T cells, which according to previous reports was mediated by CAF‐related secretome.^[^ ^33^, ^34^ ^]^ Combining all mechanistic evidences, we summarized ANO1's functional details in GI cancer microenvironment: through inhibiting cancer cell ferroptosis in a PI3K‐Akt‐dependent manner, ANO1 stimulates the production and secretion of TGF‐*β* by cancer cells, subsequently strengthens CAF recruitment, and cripples CD8^+^ T cell‐mediated anti‐tumor immune responses, generating resistance to immunotherapy (Figure 8T).

Furthermore, we realized that talabostat augmented immunotherapy in CT26 CDX, while the triple‐combination of ANO1 knockdown/anti‐PD‐1 antibody/FAP inhibitor maximized anti‐tumor effectiveness comparably with the double‐combination of ANO1 knockdown/FAP inhibitor or the double‐combination of ANO1 knockdown/anti‐PD‐1 antibody (Figure 8U). Since we have proved ANO1 as an upstream promoter of CAF, it is reasonable that targeting CAF was dispensable to overcome immunotherapeutic resistance under the presence of ANO1 inhibition. Altogether, these data emphasized that ANO1‐mediated ferroptosis inhibition of cancer enhances CAF accumulation in TIME, which at least partially contributes to ANO1‐induced immunotherapeutic resistance.

## 3 Discussion

GI cancers possess high malignancy, complicated molecular composition, and highly heterogenic TIME, thus they lack universal therapeutic targets and generally display inactivate anti‐cancer immunity. For now, immunotherapy that targeting PD‐1/PD‐L1 has been one of the most promising treatment options, yet its effectiveness against GI cancers remains unsatisfying, partially because of the incidence rate of profitable predictors (majorly CPS and MSI/dMMR) cover only a small proportion of populations and display imperfect or even controversial indications in directing clinical decision‐makings.^[^ ^35^ ^]^ Additionally, a number of patients also developed acquired resistance and failed to continuously benefit from immunotherapy.^[^ ^36^ ^]^ Besides immune checkpoints, HER2, EGFR, and angiogenesis pathway members are the few molecular targets applicable for approved regimens against GI cancers, which severally restricted personalized therapeutic options.^[^ ^37^, ^38^ ^]^ As a result, it is crucial to explore more therapeutic targets for GI cancers and find new routes to overcome resistance to current immunotherapy.

Through analyzing a retrospective GI cancer cohort collected by our group, we noticed that among all CNAs with >5% incidence rate, ANO1 was the only gene that amplified completely in immunotherapeutic nonresponders. Patients harboring ANO1 amplification/hyperexpression displayed significantly shortened irPFS/irOS, which was observed in other retrospective GI cancer cohorts and a melanoma cohort. According to two observational trials, IHC‐based ANO1 positivity at baseline also prospectively predicted adverse immunotherapeutic outcomes of GI cancers. To our interest, although ANO1 was frequently amplified/upregulated in GI cancers, neither its copy number nor its expression shared sufficient correlation with MSI/CPS as well as other genetic or expressional traits as biomarkers/targets for current precision treatment. This unique distribution of ANO1 in GI cancers highlighted its importance as an independent predictor for immunotherapy and a potential therapeutic target.

Under in vitro or immunodeficient in vivo conditions, ANO1 knockdown evidently suppressed GC's proliferation/migration/invasion and peritoneal/liver/lung metastasis, suggesting ANO1 itself plays a cancer promotive role independent of the immune system, which might be due to ANO1's function in activating PI3K‐Akt signaling and inhibiting ferroptosis in cancer cells. In addition to knockdown, we noticed two ANO1‐targeted inhibitors targeting effectively repressed the growth of GI cancer cell lines/CDXs as well as PDXs. In immune‐intact mice CDXs, apart from inhibiting tumor growth, ANO1 knockdown also increased the anti‐tumor immune activity, marked by increased infiltration/expression of CD8^+^ T cells/IFN‐*γ*/TNF‐*α*/granzyme B/IL‐13 and reduced PD‐L1 expression. These data were in accordance with the result from mIHC and TCGA analysis, suggesting that ANO1 contributed to an immune‐suppressive tumor microenvironment by inhibiting the accumulation of cytotoxic CD8^+^ T cells. Consistent with our findings in patient cohorts, MC38 xenograft displayed elevated ANO1 expression after acquired immunotherapeutic resistance, while ANO1 knockdown or inhibitors augmented the effectiveness of anti‐PD‐1 antibody against mouse GI cancer CDXs harboring primary sensitivity, primary resistance, and acquired resistance to immunotherapy. These data proved that ANO1 served not only as a valuable target for GI cancers’ monotherapy, but also could be an option for combination strategy to re‐sensitize current immunotherapy regimens.

Mechanistically, we found ANO1 exerted its malignant functions in promoting tumor growth/metastasis and mediating immunotherapeutic resistance through inhibiting cancer ferroptosis, an iron‐regulated cell death triggered by the accumulation of toxic lipid peroxides. Tumor cells usually harness specific mechanisms to evade ferroptosis and to facilitate tumor progression,^[^ ^39^ ^]^ while ferroptosis is also frequently prevented in other immune suppressive cells of TIME, such as Tregs and myeloid–derived suppressor cells.^[^ ^40^ ^]^ Importantly, CD8^+^ T cells repress the expression of system Xc^−^ in tumor cells through releasing IFN‐*γ*, thus provoking lipid peroxidation and inducing ferroptosis in tumor cells, contributing to their anti‐tumor effects.^[^ ^41^ ^]^ In turn, ferroptotic cancer cells can release immunostimulating signals, notably damage‐associated molecular pattern signals (e.g., HMGB1/calreticulin/DNA/ATP) that attract antigen presenting cells, promoting dendritic cell maturation, increasing the efficiency of macrophage‐mediated phagocytosis of cancer cells, and enhancing the CD8^+^ T cell infiltration in tumors, subsequently enhancing tumor‐specific immune responses as well as the efficacy of immune checkpoint inhibitors.^[^ ^42^, ^43^, ^44^, ^45^ ^]^ ANO1 intercepting the endogenous ferroptosis machinery avoided cancer cells from CD8^+^ T cell‐mediated ferroptotic cytotoxicity and lead to immune escape. On molecular level, we found ANO1 downregulates NRF2 and SLC7A11, two key regulators in inhibiting ferroptosis. Under quiescent conditions, NRF2 interacts with its binding partner KEAP1 (an adaptor of the ubiquitin ligase complex) and is constitutively degraded through the ubiquitin‐proteasome pathway.^[^ ^46^ ^]^ As a master regulator of antioxidant defense, NRF2 subsequently promotes the transcription of many ferroptosis suppressor genes, including SLC7A11.^[^ ^47^, ^48^ ^]^ SLC7A11 is a crucial part of the cystine uptake channel system Xc^−^, which governs the SLC7A11–GSH–GPX4 axis and constitutes the major cellular system defending against ferroptosis.^[^ ^48^, ^49^ ^]^

We further proved that ANO1 upregulates NRF2/SLC7A11 expression and inhibits ferroptosis in a PI3K‐Akt signaling dependent manner. Cancer cells carrying PIK3CA activation or PTEN deletion are generally resistant to ferroptosis.^[^ ^50^ ^]^ Mechanistically, the major PI3K‐Akt signaling member mTORC1 phosphorylates p62 and promotes the interaction of p62 with the NRF2‐binding site on KEAP1, competitively inhibits the KEAP1‐NRF2 interaction, and leads to KEAP1 degradation and NRF2 accumulation, allowing stabilized NRF2 to translocate to the nucleus and inhibits ferroptosis through transcriptionally upregulating SLC7A11.^[^ ^46^, ^51^ ^]^ Oncogenic activation of PI3K‐Akt‐mTORC1 signaling also promotes the SREBP/SCD1‐mediated monounsaturated fatty acid‐containing phospholipid synthesis, reducing lipid ROS and suppressing ferroptosis.^[^ ^50^ ^]^ For the mechanistic link between ANO1 and PI3K‐Akt signaling, it has been reported that ANO1 contributes to tumorigenesis of multiple cancers by activating EGFR and CAMKII, subsequently inducing activation of AKT and MAPK signaling.^[^ ^52^ ^]^ ANO1 also increases EGFR‐protein level in cancer cells through a posttranslational, degradation‐independent mechanism, subsequently activating downstream PI3K‐Akt signaling.^[^ ^53^ ^]^ In accordance with these reports, we also found ANO1's transcript expression was positively correlated with EGFR expression for both TCGA‐STAD and ‐ESCA datasets (Figure S2I, Supporting Information). Since PI3K‐Akt is one of the most frequently mutated and activated signaling in human cancers, whether ANO1 would ignite other pathways downstream of PI3K‐Akt signaling deserved further exploration.

We also noticed that through restoring inhibited ferroptosis, ANO1 knockdown repressed cancer cells’ expression/secretion of TGF‐*β* and the recruitment of CAFs. Since ANO1 prevented cancer cells from ferroptotic death, we attributed ANO1‐facilitated on TGF‐*β* secretion and CAF recruitment as the consequence of increased cancer cell survival. CAFs are typically driven by tumor‐released factors (especially TGF‐*β*), while activated CAFs are also characterized by elevated TGF‐*β* secretion in feedback.^[^ ^54^ ^]^ CAFs influence cancer initiation/progression/metastasis/angiogenesis and immune responses through its secretome or physical interaction with tumor cells.^[^ ^55^ ^]^ Moreover, CAFs were reported to form a sphere that surrounds and protects cancer cells from immune cytotoxicity.^[^ ^28^ ^]^ CAFs can also result in TIME remodeling by upregulating immune checkpoints (such as PD‐L1/CTLA‐4) on T cells to induce immune exhaustion,^[^ ^56^ ^]^ which was in concert with our findings that ANO1 reduced CD8^+^ T infiltration and anti‐cancer immune cytotoxicity while targeting ANO1/CAF augmented the effectiveness of immunotherapy. Collectively, our work proved that the amplification/upregulation of ANO1 in GI cancers abolishes cancer ferroptosis in a PI3K‐Akt signaling‐dependent manner, consequently increases cancer cell survival and strengthens CAF recruitment through elevating TGF‐*β* release, and thus promotes tumor progression and cripples anti‐tumor immune responses, generating resistance to immunotherapy.

Considering ANO1's critical role in regulating cancer progression and immune escape, we recommend it both as a feasible biomarker for immunotherapy and a promising target for future therapies. As mentioned above, ferroptosis plays important role in regulating microenvironment and anti‐tumor immunity, thus the targeted induction of ferroptosis could be a new strategy for anti‐cancer treatment. However, the overlap of ferroptotic machinery among tumor cells, tumor‐promotive TIME cells, and tumor‐suppressive TIME cells confers similar vulnerabilities, provoking indiscriminate lipid peroxidation and cell death in the microenvironment and making it difficult to therapeutically harness tumor vulnerability without affecting anti‐tumor immunity. On the other hand, therapeutic approaches targeting CAFs has also been assessed in full swing by ongoing preclinical and clinical studies. Since ANO1 is highly expressed in cancer tissue, exploiting its inhibition can induce ferroptosis selectively on cancer cells rather than on immune cells while suppressing accumulation/function of CAFs in TIME. Thus, targeting ANO1 is expected to produce remarkable effect through simultaneously harnessing PI3K‐Akt inhibition, ferroptosis induction, and CAF inhibition. Moreover, since ANO1 was highly expressed in various cancer types other than GI cancers, it may hopefully be a pan‐cancer immunotherapy biomarker and a target for precision treatment. Since we have proved two ANO1 inhibitors (CAI/BBR) were feasible for GI cancer cell lines/CDXs/PDXs, their safety and effectiveness in clinical applications deserved to be investigated, and other inhibitors with higher ANO1‐targeted specificity and tumor killing effectiveness should also be developed by future studies.

In summary, our work demonstrated comprehensive evidences to ANO1's role in GI cancers. Through unveiling the existence of an ANO1‐PI3K‐Akt‐ferroptosis‐CAF regulative axis, our work outlined the functional details of ANO1 in mediating tumor progression and TIME remodeling, provided clues for GI cancers’ low effectiveness/resistance to immunotherapy, and introduced ANO1 as a promising target for anti‐cancer precision treatment.

## 4 Experimental Section

### Ethics Approval and Consent to Participate

All trials/specimens/associated clinical information were approved for research applications by the Institutional Ethics Committee of Peking University Cancer Hospital and Institute (2020KT08). Written informed consents were obtained from all providers. This study was conducted in accordance with the Declaration of Helsinki. All animal experiments were performed under the permission of Animal Experimentation Ethics Committee of Peking University Cancer Hospital (EAEC 2021‐01).

### Immunotherapy Cohorts of GI Cancer Patients

Patients received immunotherapy‐related regimens (anti‐PD‐1/anti‐PD‐1 along or plus anti‐CTLA4/chemotherapy) were enrolled from conventional treatments or clinical trials hosted by the Department of Gastrointestinal Oncology, Peking University Cancer Hospital and Institute. Enrolled patients must be diagnosed with certain type of GI cancers (including cancers originated from stomach/esophagus/intestine/liver/pancreas/bile duct), remained treatment naive 3 weeks before immunotherapy baseline, and with Eastern Cooperative Oncology Group score = 0/1, 3 days before treatment baseline.

99 GI cancer patients (excluding GIST) treated during January 2016 to August 2020 were used as a retrospective training cohort, 48 GI cancer patients (excluding GIST) treated during November 2018 to January 2021 from two observational studies (NCT04993378/NCT05427227) were used as a prospective validating cohort, while four GIST patients treated during April 2018 to January 2022 were used for independent analysis. All clinical data were acquired by referring to patients’ individual medical records. Based on irRECIST criteria,^[^ ^57^ ^]^ patients’ responses to therapies were evaluated as complete response (CR)/partial response (PR)/stable disease (SD)/progressed disease (PD). Objective response rate, disease control rate, irPFS, and irOS were calculated. During the whole follow‐up, patients who reached CR/PR, or SD ≥ 6 months were defined as responders, while other patients were defined as nonresponders. Progression or death of responders was defined as acquired resistance.

### Availability of Other Specimens and Datasets

TCGA (https://portal.gdc.cancer.gov/), GSE62254/GSE51105/GSE78220 (https://www.ncbi.nlm.nih.gov/geo/), and MSK datasets (https://www.cbioportal.org/) were free for use. Patient tissue for formalin‐fixed and paraffin‐embedded (FFPE) slides or PDXs was collected by the Department of Gastrointestinal oncology and Department of Pathology, Peking University Cancer Hospital and Institute. Patient tissue microarray was purchased from Outdo Biotech Co., Ltd. (Shanghai, China).

### Cell Lines

Human GC cell lines AGS/HGC27, human EC cell lines KYSE‐70/KYSE‐150/KYSE‐180/KYSE‐450/KYSE‐510, human CRC cell lines HCT116/LOVO/HT29, and mouse COAD cell lines MC38/CT26 were from ATCC (Manassas, VA) or National Infrastructure of Cell Line Resource (Beijing, China), and were authenticated by short tandem repeat profiling. Cells were maintained in RPMI1640/DMEM (Invitrogen) medium supplemented with 10% fetal calf serum (Gibco, BRL) and 1% penicillin plus streptomycin (HyClone, Logan, UT).

### Gene Interference and Overexpression

Lentiviral overexpression system of human‐ANO1 was provided by Genechem Biotech (Shanghai, China) based on a GV705 plasmid. ANO1 interference system was provided by Sangon Biotech (Shanghai, China). Interference sequences: human sh‐1‐ANO1: 5′‐GGTTCCCAGCCTACCTCACT‐3′, 5′‐GAGATTCTGTAGATGATGACGCC‐3′; human sh‐2‐ANO1: 5′‐TCTTCCTTCAATGTCAGCGACT‐3′, 5′‐GGTTCCCGGTAATCTTTATACCT‐3′; mouse sh‐1‐ANO1: 5′‐CCGGGTTCTCATGGTGGAGCTGTTTCTCGAGAAACAGCTCCACCATGAGAACTTTTTTG‐3′; 5′‐aattcaaaaaaGTTCTCATGGTGGAGCTGTTTCTCGAGAAACAGCTCCACCATGAGAAC‐3′; sh‐2‐ANO1: 5′‐CCGGCCAAATACTGAGAGGAAATTACTCGAGTAATTTCCTCTCAGTATTTGGTTTTTTG‐3′; 5′‐aattcaaaaaaCCAAATACTGAGAGGAAATTACTCGAGTAATTTCCTCTCAGTATTTGG‐3′.

For transfection, 2 mL of cells (5 × 10^4^/mL) were inoculated into a 6‐well plate for 24 h. Lentivirus’ volume for transfection was calculated based on the formulate: volume (mL) = MOI × number inoculated of cells/viral titer (TU mL^−1^). Polybrene was used to facilitate transfection. After 24 h transfection, the culture medium was refreshed.

For the selection of stable clones, complete culture medium containing 2 µg mL^−1^ of puromycin (InvivoGen, San Diego, CA, USA) was added into culture wells to construct stably infected cell lines. Protein expression level of the target genes were appraised by immunoblot assay after 2 weeks.

### Antibodies, Reagents, and Dosing

For antibodies, ANO1 (A10498)/SLC7A11 (A2413)/NRF2 (A0674) were from ABclonal. GAPDH (#2118)/PDGFR‐*α* (#3174)/PDGFR‐*β* (#3169)/*α*‐SMA (#19245)/SMAD2/3 (#8685)/S100A4 (#13018)/PI3K (#4257)/p‐PI3K (Y199, #4228)/Akt (#4691)/p‐Akt (S473, #9271)/S6 (#2217)/p‐S6 (S235/236, #4858)/CD8*α* (#98941)/Ki67 (#12202, #62548)/CD31 (#77699)/PD‐L1 (#64988)/granzyme B (#46890) were from Cell Signaling Technology. FAP (ab218164)/TGF‐*β* (ab215715) were from Abcam. ANO1 (ZM‐0371)/CD31 (ZA‐0568) were from ZSGB‐BIO.

For reagents, CAI (HY‐100611)/BBR (HY‐B1135)/erastin (HY‐15763)/ferrostatin‐1 (HY‐100579)/liproxstatin‐1 (HY‐12726)/dactolisib (BEZ235, HY‐50673)/talabostat mesylate (HY‐13233A) were from MedChemExpress (Monmouth Junction, NJ, USA). Anti‐mouse‐PD‐1 blocking antibody (BE0273) was from BioXCell. Isotype control antibody Mouse IgG (SP031) was from Solarbio (Beijing, China). Active recombinant mouse TGF‐*β*1 (RP01167) was from Abclonal (Beijing, China).

*Reagents dosing*: In vitro, erastin, 30 µm for 24 h; ferrostatin‐1, 60 nm for 24 h; dactolisib, 100 nm for 24 h. in vivo, anti‐mouse‐PD‐1, 3 mg kg^−1^, twice a week for 3 weeks, intraperitoneal injection; CAI, 50 mg kg^−1^, once every other day for 3 weeks, intraperitoneal injection; BBR, 50 mg kg^−1^, once every other day for 3 weeks, oral; talabostat mesylate, 10 µg per mouse, twice 1 day for 2 weeks, oral; liproxstatin‐1, 10 mg kg^−1^, once a day for 2 weeks, intraperitoneal injection; TGF‐*β*1, 25 ng kg^−1^, once every other day for 2 weeks, intratumoral injection.

### Cell Viability Assay and Apoptosis Assay

For viability assay, cells were precultured into 96‐well plates (3 × 10^3^ per well) as triplicates, then treated with CCK‐8 kit (Dojindo laboratories, Tokyo, Japan). For apoptosis assay, digested cells were resuspended with PBS, double‐stained with Annexin V‐PE/7‐AAD, then assessed using an apoptosis detection kit (Dojindo).

### Migration and Invasion Assay

Cells resuspended with serum‐free medium (2 × 10^5^/mL, 150 µL) were inoculated in upper chamber of each transwell (pre‐coated with matrigel in terms of invasion assay) (Corning, New York, NY), cultured in complete medium for 24–48 h, then fixed with methanol and dyed with 0.1% crystal violet. Cells in upper chamber were removed with cotton wool. Penetrated cells were counted under microscope.

### Immunohistochemistry Assay

FFPE tissue slides were deparaffinized with dimethylbenzene, rehydrated with ethanol, treated with 3% H_2_O_2_ to quench endogenous peroxidase activity, boiled in EDTA solution (pH = 9.0, ZSGB‐BIO) for antigen retrieval, blocked from nonspecific bindings by 5% goat serum, and treated with haematoxylin–eosin (Solarbio) or antibody staining. Staining was scored by two pathologists by defining −/+ as negative and ++/+++ as positive.

### Immunoblot Assay

Blotted cell/tissue lysate was transferred onto polyvinylidene fluoride membranes (Millipore, USA), probed with appropriate antibodies, illuminated with Clarity Western ECL substrate (Bio‐Rad, Hercules, CA), visualized with Amersham Imager 600 (GE Healthcare, Chicago, IL), and quantified with ImageJ software.

### ELISA

The supernatant of cell/tissue lysate or mice serum were assessed with ELISA. Kits for human‐TGF‐*β*1 (#1117102)/mouse‐TNF‐*α* (#1217202)/mouse‐IFN‐*γ* (#1210002)/mouse‐IL‐4 (#1210402) were from Dakewe Bio‐engineering Co., Ltd. (Beijing, China). Kits for mouse‐TGF‐*β*1 (#CME0020)/mouse‐IL‐13 (#CME0009)/mouse‐granzyme B (#CME0057) were from 4A Biotech Co., Ltd. (Beijing, China). Targeted concentrations were calculated based on the linear range of manufacturer's standard control.

### Measurement of Ferroptosis

The MDA assay kit (BC0020‐50T/48S) was from Solarbio. The liperfluo kit (L248) was from Dojindo. All measurements were performed according to manufacturer's instructions.

### Immunofluorescent Staining of Tissue

FFPE tissue slides (4 µm) were blocked with 5% bovine serum albumin, incubated with primary antibodies overnight at 4 °C, incubated with secondary antibodies (Alexa Fluor 488‐conjugated goat anti‐rabbit IgG (A11034, Invitrogen) and Alexa Fluor 555‐conjugated goat anti‐mouse IgG (A21422, Invitrogen) at room temperature in dark, stained with DAPI (Sigma‐Aldrich, St. Louis MO, USA), and pictured with an LSM780 confocal microscope (Zeiss).

### Multiplex IHC Labeling of Tissue

Dehydrated FFPE tissue slides (4 µm) were deparaffinized/rehydrated, heated for antigen retrieval, blocked, and incubated with primary antibodies and horseradish peroxidase‐conjugated secondary antibodies. Antibody stripping and antigen retrieval were performed after each round of tyramine signal amplification. Nuclei were stained with DAPI. Stained slides were scanned using the Mantra quantitative pathology imaging system (PerkinElmer, Waltham, MA, USA) for bright field and fluorescent images, then supervised by two pathologists with Phenochart software (PerkinElmer) to select the representative region of interest and the signal intensity was measured.

### RNA Sequencing

Total RNA was extracted from cells using Trizol method. Quality control was performed using an Agilent 2100 Bioanalyzer (Agilent Technologies) to ensure RNA integrity. Next generation RNA‐sequencing was performed using an Illumina HiSeq instrument (Illumina, San Diego, CA).

### Single‐Cell RNA Sequencing

Fresh tissue was washed with Hanks balanced salt solution, minced into small pieces, digested with sCelLive Tissue Dissociation Solution (Singleron), filtered through a 40‐micron sterile strainer, centrifuged (300 × *g*, 4 °C) to remove supernatant, resuspended with PBS/DMEM, then stained with trypan blue to microscopically evaluated cell viability. Single‐cell suspensions (2 × 105 cells/mL) were loaded onto microwell chip using the Singleron Matrix Single Cell Processing System. Barcoding beads were subsequently collected from the microwell chip, followed by reverse transcription of captured mRNA. Obtained cDNA was amplified by PCR, then fragmented and ligated with sequencing adapters. The scRNA‐seq libraries were constructed by using the GEXSCOPE Single Cell RNA Library Kits (Singleron). Individual libraries were pooled and sequenced on Illumina novaseq 6000 with 150 bp paired end reads. Cell clusters were scored based on specific gene signatures, including cancer cell (*KRT10*, *KRT14*, *SOX4*, *STMN1*, *Ki67*, *SPARC*), lymphocyte (*CD2*, *CD3D/E/G*, *TRAC*, *TRBC1*, *KLRD1*, *NKG7*), myeloid cell (*LYZ2*, *LY6C2*, *CCR2*, *C1QC*, *MRC1*, *CD68*, *CD209A*, *XCR1*), endothelial cell (*CDH5*, *PECAM1*, *CLDN5*, *VWF*, *KDR*), and fibroblasts (*DCN*, *LUM*, *COL1A2*, *COL1A1*). Fibroblasts were further stratified into CAFs and normal tissue‐associated fibroblasts according to scoring a CAF specific gene signature (*ACTA2*, *FAP*, *PDPN*, *VIM*, *TNC*, *FN1*, *POSTN*, *DES*, *PDGFRA*, *PDGFRB*, *S100A4*, *MMP1*, *MMP3*, *MMP9*, *COL1A1*, *COL1A2*, *TGFB1*, *TGFB3*, *COL6A1*, *FGF7*, *CXCL12*, *COL11A1*).

### Cell‐Derived Xenograft and Experiments

HGC27 cells were inoculated into 5‐week‐old female BALB‐C nude mouse (Vital River Laboratories, Beijing, China). 5 × 10^6^ cells suspended in 100/70/200 µL were injected into subcutaneous/spleen/caudal vein to assess tumorigenesis/local dissemination/distant metastasis ability, respectively. 3 × 10^6^ cells suspended in 200 µL were injected intraperitoneally to study local colonization ability. Tumor volume was measured every 3 days by the formula *V* = *L* × *W* ^2^ × 0.5 (*V*, volume; *L*, length; *W*, width of tumor). After 1–4 months injection, mice were sacrificed and dissected to observe metastasis.

CT26/MC38 cells were inoculated into BALB‐C/C57BL‐6J mice (Vital River Laboratories), respectively. When tumor volume reaches 150 mm^3^, mice were randomly grouped and treated with specific regimens. Mice were sacrificed when tumor volume over 2000 mm^3^ or after 21 days dosing period. Tumor tissue were then stripped and prepared for other assessments. MC38 CDX was treated with long term, low dose anti‐PD‐1 antibody (BE0273, BioXCell) induction to acquire resistance (MC38‐R).

### Patient‐Derived Xenograft and Experiments

Fresh tissue samples from patients were inoculated into 5‐week‐old female NOD/SCID mice (HFK Biotechnology, Beijing, China) to construct PDXs. PDXs after five passages were adopted to test ANO1 positivity and to evaluate the effectiveness of pharmaceutical reagent. GC PDX case‐TS was treated with long term, low dose trastuzumab induction to acquire resistance (case‐TR).

### Statistics Analysis and Formatting

Diversity between subgroups was assessed by chi‐square test/unpaired two‐tailed *t*‐test/one‐way or repeated‐measures ANOVA. Consistency between indexes was assessed by Pearson correlation analysis. Survival proportions were assessed by Kaplan–Meier analysis paired with Log‐rank test, or COX regression model. A two‐sided *p* < 0.05 was considered statistically significant. Analysis and graphing were powered by R 3.5.1/SPSS 21.0/GraphPad Prism 8/BioRender programs. Error bars were exhibited in form of mean ± standard error of mean (SEM).

## Conflict of Interest

The authors declare no conflict of interest.

## Supporting information

Supporting Information

## Data Availability

The data that support the findings of this study are available from the corresponding author upon reasonable request.
