# Supplementary material for: ANO1‐Mediated Inhibition of Cancer Ferroptosis Confers Immunotherapeutic Resistance through Recruiting Cancer‐Associated Fibroblasts
Source: Adv Sci (Weinh). 2023 Jun 21;10(24):2300881. doi: 10.1002/advs.202300881 (PMC10460848; doi:10.1002/advs.202300881)
Supplement: Supplementary file 1 — Supporting Information [file ADVS-10-2300881-s001.pdf]

ADVANCED SCIENCE
Open Access

Supporting Information

for *Adv. Sci.*, DOI 10.1002/advs.202300881

ANO1-Mediated Inhibition of Cancer Ferroptosis Confers Immunotherapeutic Resistance through Recruiting Cancer-Associated Fibroblasts

Fangli Jiang, Keren Jia, Yang Chen, Congcong Ji, Xiaoyi Chong, Zhongwu Li, Feilong Zhao, Yuezong Bai, Sai Ge, Jing Gao, Xiaotian Zhang, Jian Li, Lin Shen* and Cheng Zhang**

Supporting Figures and Supporting Tables

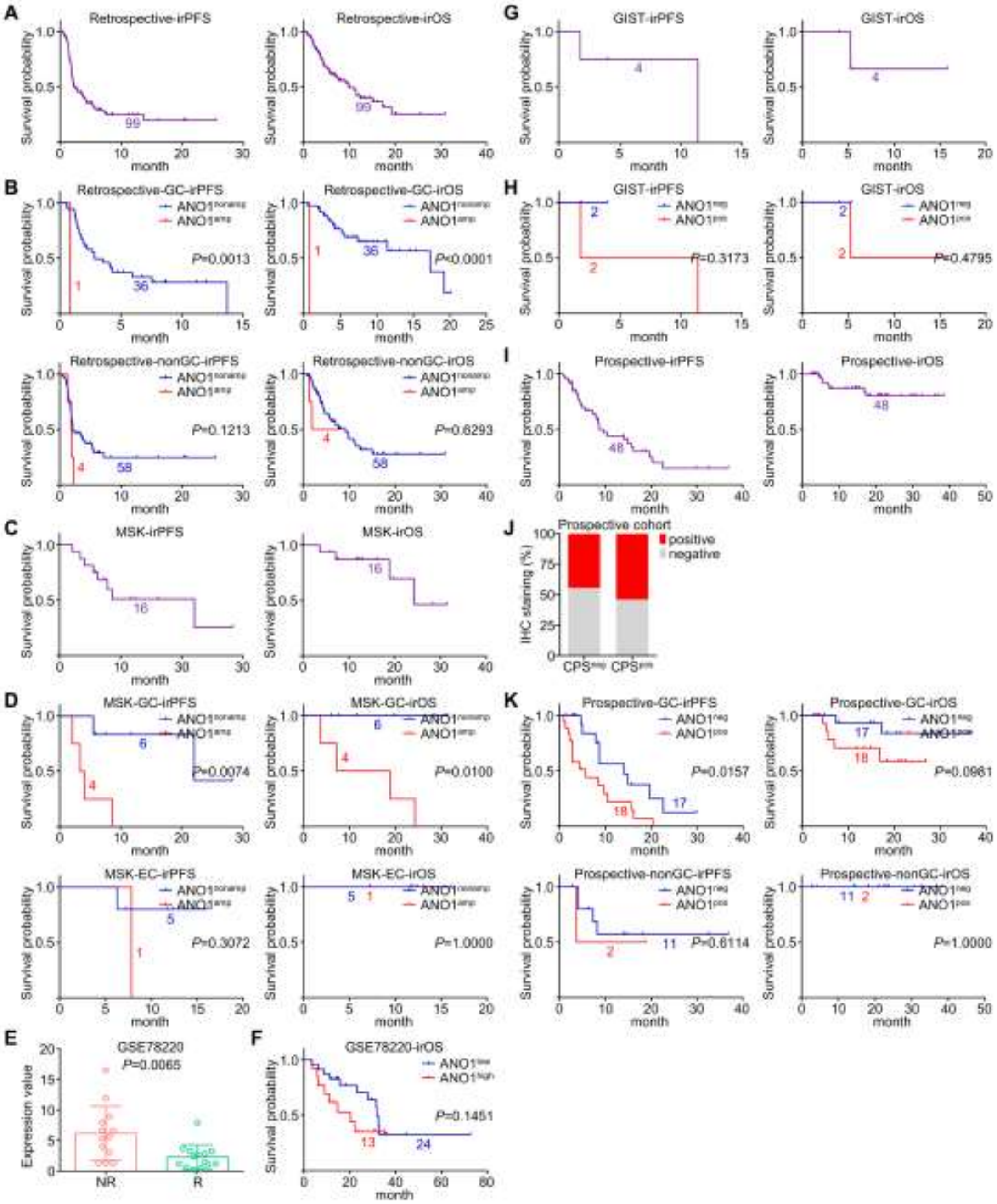

Figure S1. Further stratification for ANO1's indication to immunotherapeutic outcomes.

(A) Overall irPFS/irOS for the 99-case GI cancer retrospective cohort received immunotherapy. **(B)** The prognostic correlation of ANO1 amplification in training cohort was measured for patients of GC and EC+CRC+other cancers. **(C)** Overall irPFS/irOS for the MSK dataset. **(D)** The prognostic correlation of ANO1 amplification in MSK dataset was measured for patients of GC and EC. **(E)** The immunotherapeutic responses and **(F)** irOS for melanoma GSE78220 cohort were stratified by ANO1 expression. **(G)** Overall irPFS/irOS for four GIST patients received immunotherapy. **(H)** irPFS/irOS for GIST patients were stratified by IHC-based ANO1-positivity. **(I)** Overall irPFS/irOS for the 48-case GI cancer prospective cohort received immunotherapy. **(J)** Comparison of IHC-based ANO1 positivity between CPS-negative (CPS=0) and -positive (CPS≥1) patients in the prospective cohort. **(K)** The

prognostic correlation of ANO1-positivity in the prospective cohort was measured for patients of GC and EC+CRC+other cancers.

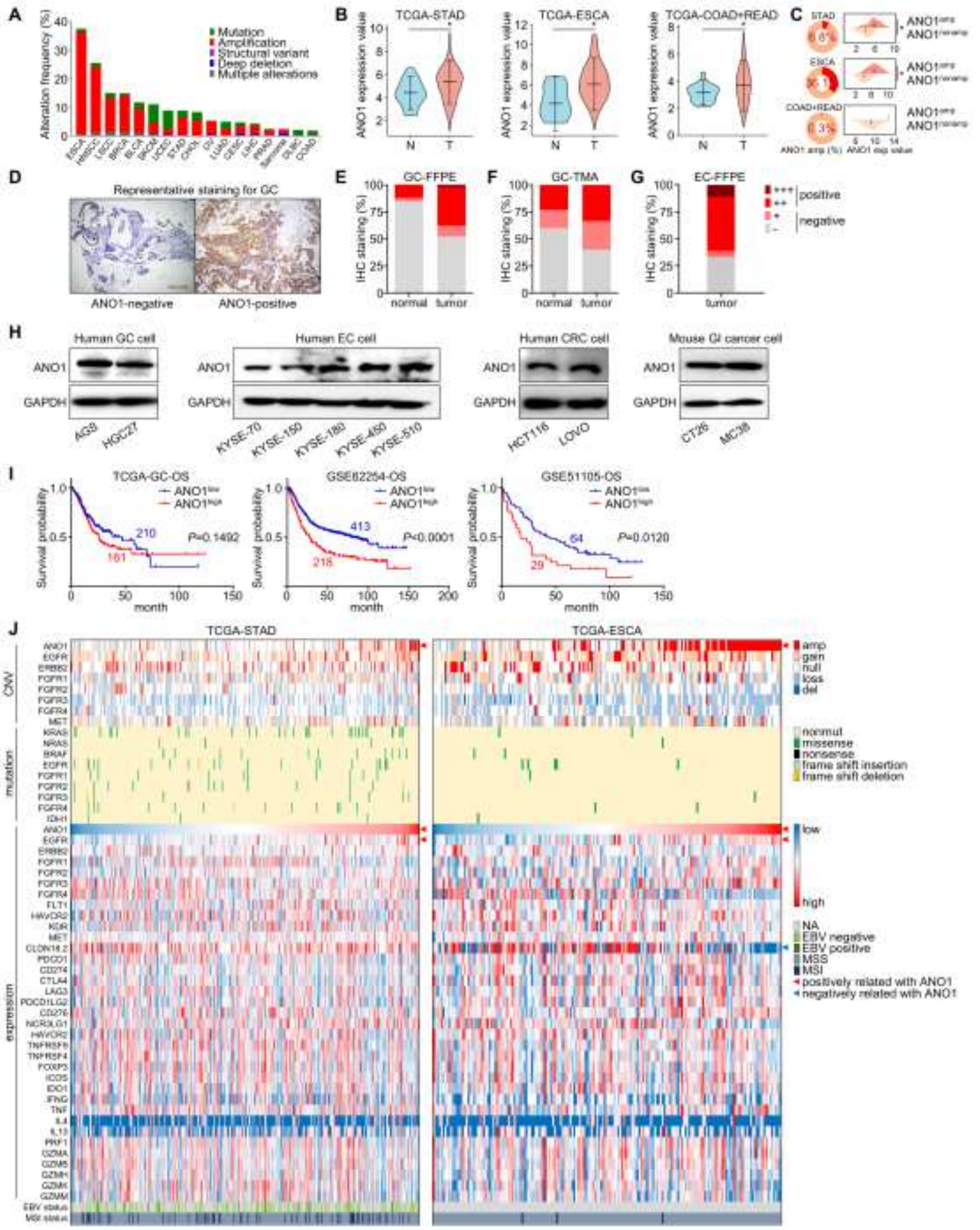

Figure S2. The genetic and expressional landscape of ANO1 in GI cancers.

(A) ANO1's genetic aberrance across TCGA cancer types. **(B)** Transcript levels of ANO1 in tumor (T) vs normal (N) tissues of TCGA-STAD/ESCA/COAD+READ datasets. **(C)** Transcript levels of ANO1 in ANO1 amplified vs nonamplified tumors of TCGA-STAD/ESCA/COAD+READ datasets. **(D)** Representative imaging of ANO1 IHC staining in GC tissues. Positivity rate of ANO1 in **(E)** a surgery GC cohort, **(F)** a tissue microarray (TMA) GC cohort and **(G)** an EC cohort. **(H)** ANO1 expression in multiple human and mouse GI cancer cells. **(I)** The prognostic correlation of ANO1 with OS in multiple GC datasets. **(J)** ANO1's distribution with major therapeutic biomarkers & targets of GI cancers in TCGA-STAD/ESCA datasets. *, P<0.05. Error bars, mean ± SEM.

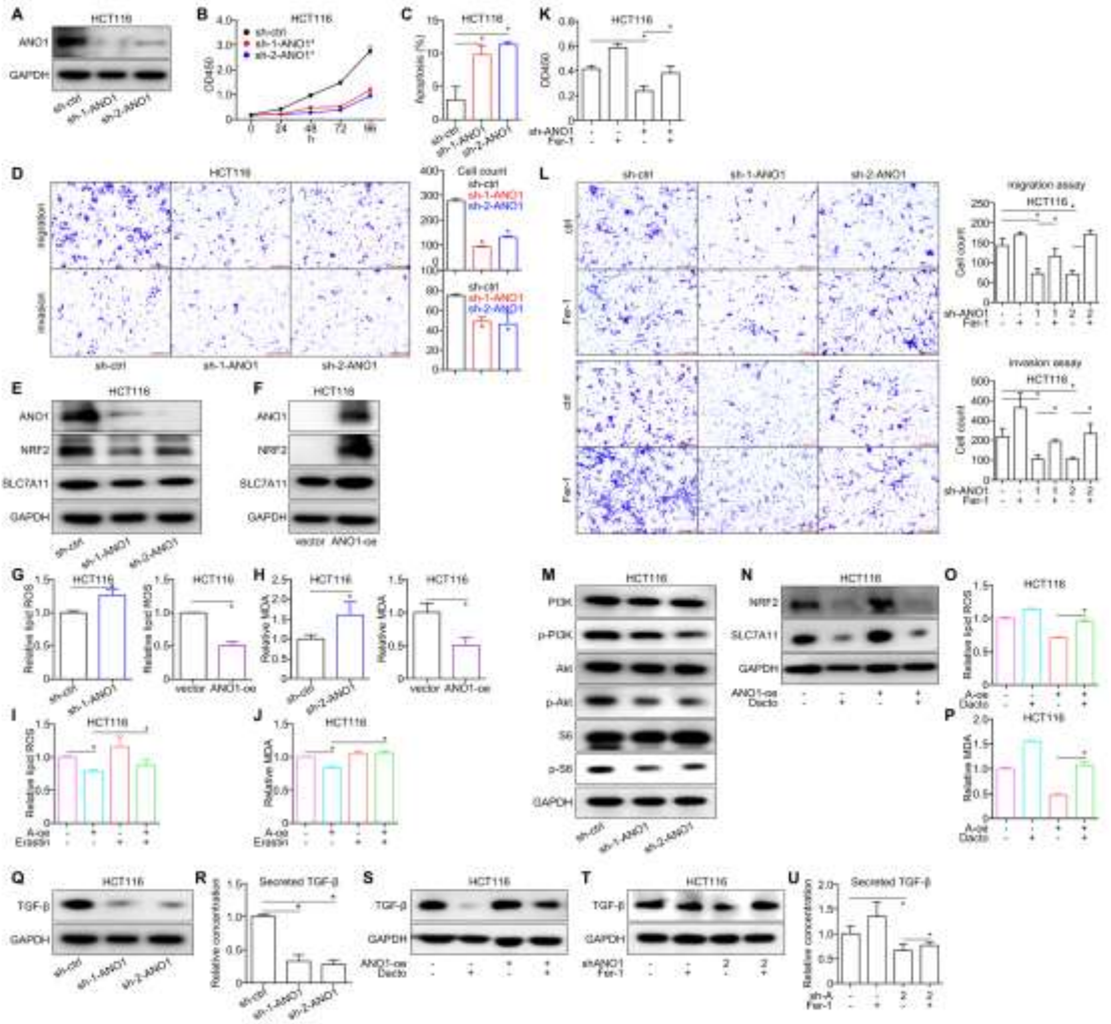

Figure S3. The *in vitro* phenotypes & mechanisms of ANO1 were consistently observed for CRC cell.

(A) ANO1 knockdown in CRC cell. Changes of *in vitro* **(B)** proliferation, **(C)** apoptosis and **(D)** invasiveness were measured after ANO1 knockdown. Expression of ferroptotic proteins after **(E)** ANO1 knockdown or **(F)** overexpression (oe) were assessed in CRC cell. The levels of **(G)** lipid ROS and **(H)** MDA after ANO1 knockdown or overexpression were assessed. **(I)** Lipid ROS and **(J)** MDA repressed by ANO1 overexpression was rescued by ferroptosis agonist Erastin in CRC cell. The *in vitro* **(K)** proliferation and **(L)** invasiveness suppressed by ANO1 knockdown were reversed by ferroptosis inhibitor Fer-1 in HCT116 cell. **(M)** ANO1 knockdown in CRC cell deactivated PI3K-Akt signaling. PI3K-Akt signaling inhibitor Dactolisib (Dacto) abrogated **(N)** the NRF2/SLC7A11 upregulation, rescued the **(O)** lipid ROS/**(P)** MDA inhibition. The **(Q)** expression and **(R)** secretion of TGF-β by CRC cell HCT116 were assessed after ANO1 knockdown. **(S)** PI3K-Akt signaling inhibitor Dactolisib (Dacto) repressed the TGF-β upregulation induced by ANO1 overexpression. The **(T)** expression and **(U)** secretion of TGF-β by CRC cell were reduced by ANO1 knockdown and rescued by ferroptosis inhibitor Fer-1. *, P<0.05. Error bars, mean ± SEM.

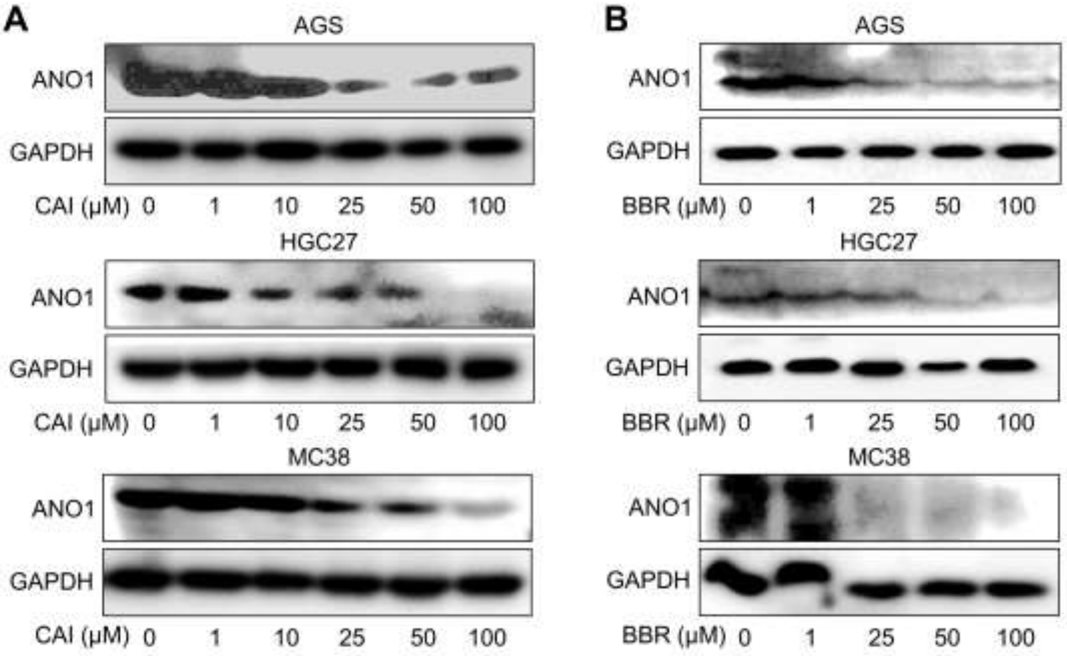

Figure S4. ANO1 inhibitors repressed ANO1 expression in a dose-dependent manner.

Changes of ANO1 protein expression in multiple GI cancer cells as the dose of ANO1 inhibitors **(A)** CaCCinh-A01 or **(B)** Benzbromarone escalating.

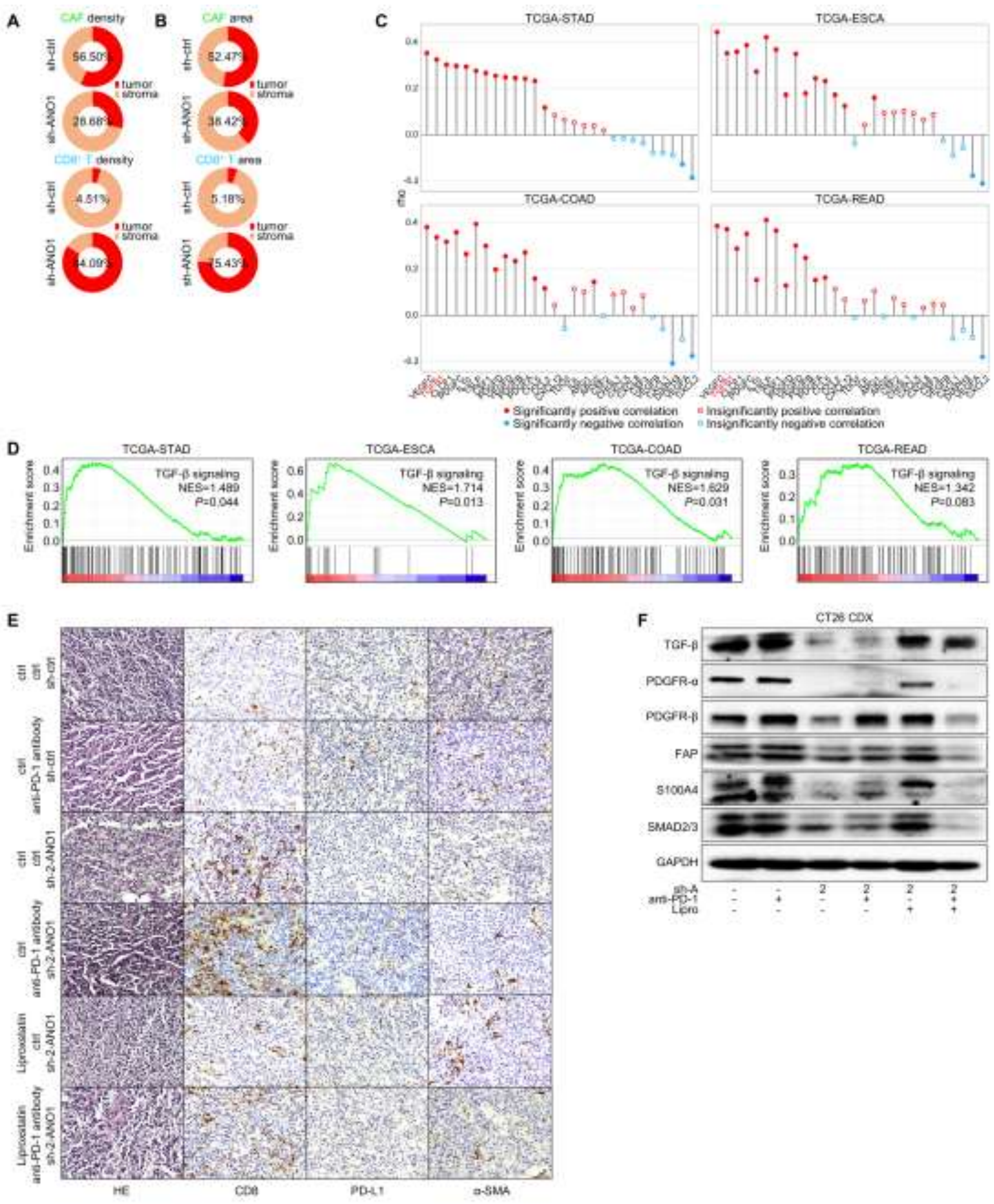

Figure S5. ANO1 promotes CAF infiltration in TIME and facilitates the CAF secretome.

The **(A)** density ratio and **(B)** area ratio of CAFs and CD8^{+} T cells in tumor/stroma regions were assessed by mIHC analysis. **(C)** ANO1 expression displayed a high correlation with CAF secretome members across TCGA-STAD/ESCA/COAD/READ datasets. **(D)** ANO1-related enrichment of TGF-β signaling in TCGA-STAD/ESCA/COAD/READ datasets. **(E)** IHC staining for infiltrated CD8/PD-L1/α-SMA in CT26 xenograft tissue. **(F)** Immunoblot-based expression of TGF-β and CAF markers in CT26 xenograft tissue.

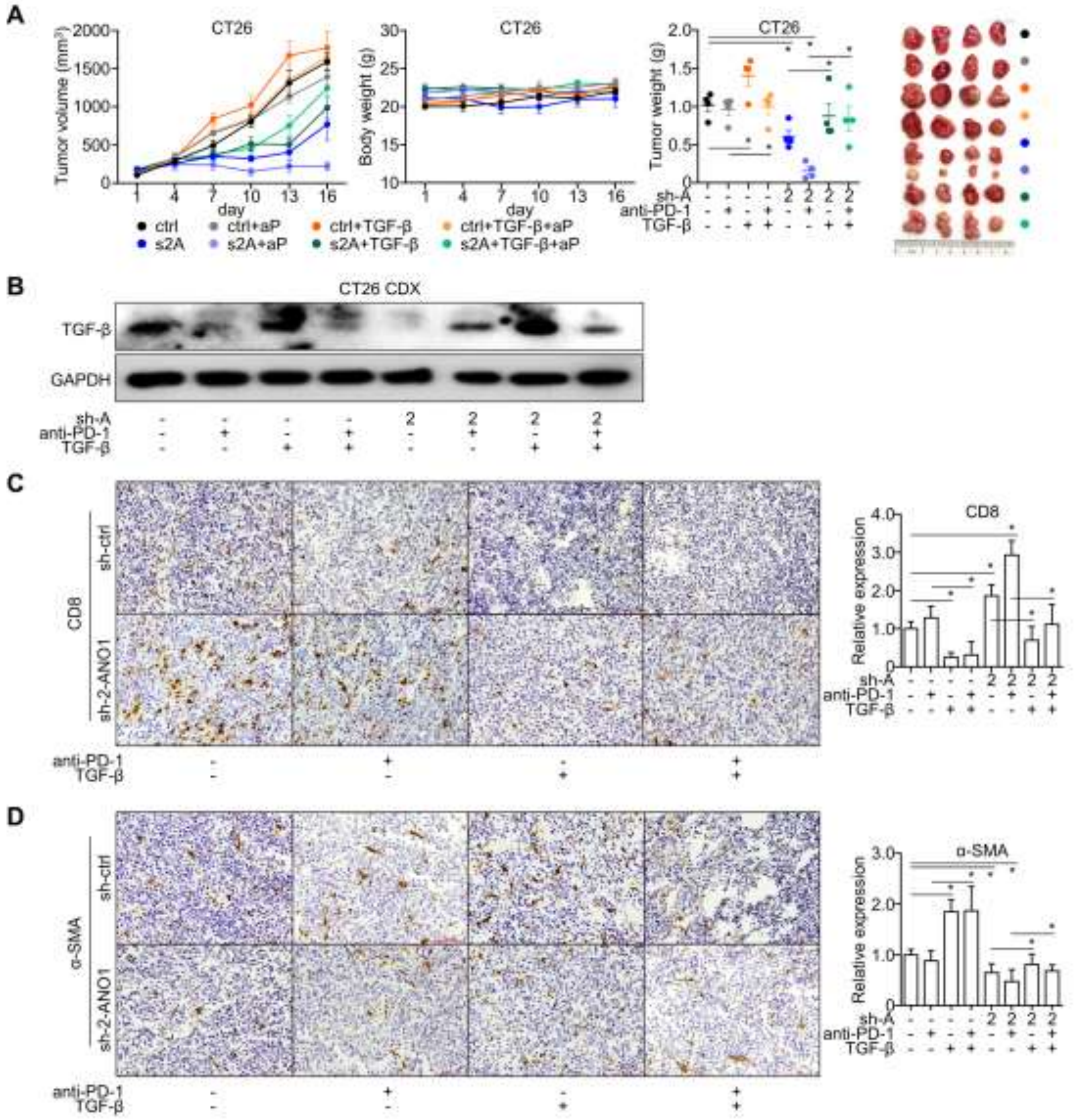

Figure S6. The impact of ANO1-PI3K-Akt-ferroptosis axis on immunotherapy efficacy, CD8⁺ T cell infiltration and CAF recruitment depends on TGF-β.

(A) The sensitized anti-tumor effectiveness in CT26 CDX by combining ANO1 knockdown and anti-PD1 antibodies was alleviated by TGF-β. **(B)** The expression of TGF-β in CT26 CDX tissues were measured by immunoblot. $CD8^+$ T cell infiltration and CAF recruitment in CT26 CDX tissues were measured by staining **(C)** CD8 and **(D)** α-SMA with IHC. *, *P*<0.05. Error bars, mean ± SEM.

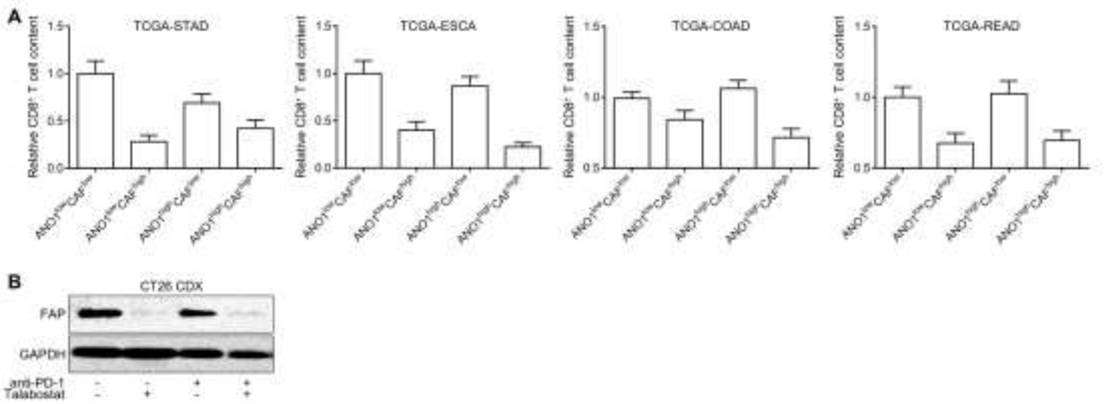

Figure S7. The relationship among CD8$^+$ T cells, ANO1 and CAFs in GI cancers.

(A) The content of CD8$^+$ T cells in TCGA-STAD/ESCA/COAD/READ datasets were stratified by ANO1 expression and CAF content. **(B)** The effect of Talabostat mesylate combined with anti-PD-1 antibodies on inhibiting FAP expression in CT26 CDX tissue. Error bars, mean ± SEM.

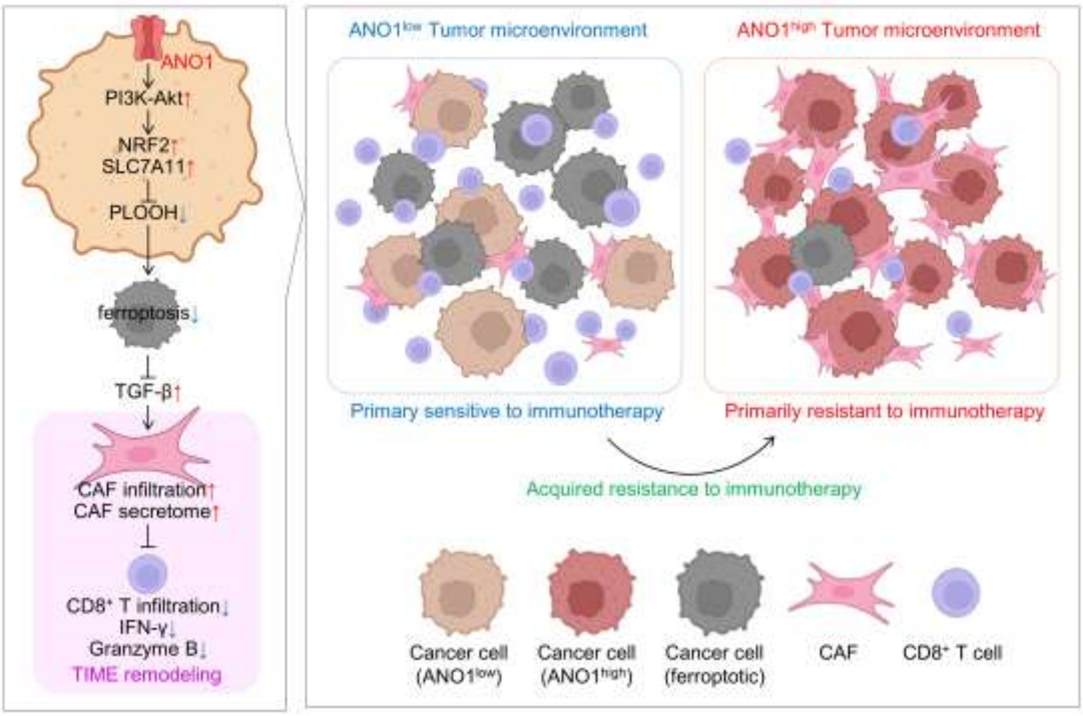

Schematic chart

Table S1. Demographic features of the retrospective training cohort classified by ANO1 status.

99-case training cohort		ANO1 status (by DNA sequencing)		
n (%)		Non-amplification	Amplification	*P* value
Total		94	5	
Gender				0.673
	male	67 (71.3)	4 (80.0)	
	female	27 (28.7)	1 (20.0)	
Age				0.597
	≥60	49 (52.1)	2 (40.0)	
	<60	45 (47.9)	3 (60.0)	
Tumor type				0.054
	Esophageal cancer	23 (24.5)	4 (75.0)	
	Gastric cancer	36 (38.3)	1 (25.0)	
	Colorectal cancer	21 (22.3)	0 (0.0)	
	Others	14 (14.9)	0 (0.0)	
Stage				0.683
	I	0 (0.0)	0 (0.0)	
	II	0 (0.0)	0 (0.0)	
	III	3 (3.2)	0 (0.0)	
	IV	90 (95.7)	5 (100.0)	
	n/a	1 (1.1)	0 (0.0)	
HER2 positivity				1.000
	positive	4 (4.3)	0 (0.0)	
	negative	50 (53.2)	2 (40.0)	
	n/a	40 (42.6)	3 (60.0)	
CPS				0.245

0	29 (30.9)	0 (0.0)	
≥1	32 (34.0)	3 (60.0)	
n/a	33 (35.1)	2 (40.0)	
MSI/MMR status			0.550
MSI/dMMR	22 (23.4)	0 (0.0)	
MSS/pMMR	49 (52.1)	3 (60.0)	
n/a	23 (24.5)	2 (40.0)	
Regimen			0.422
anti-PD-1/PD-L1	81 (86.2)	5 (100.0)	
anti-PD-1/PD-L1 plus anti-CTLA4	9 (9.6)	0 (0.0)	
anti-PD-1/PD-L1 plus chemotherapy	3 (3.2)	0 (0.0)	
anti-PD-1/PD-L1 plus targeted therapy	1 (1.1)	0 (0.0)	
anti-PD-1/PD-L1 plus chemotherapy plus targeted therapy	0 (0.0)	0 (0.0)	
Therapeutic line			0.525
1	36 (38.3)	1 (20.0)	
2	38 (40.4)	2 (40.0)	
3 or higher	19 (20.2)	2 (40.0)	
n/a	1 (1.1)	0 (0.0)	
Best response			0.102
CR/PR	22 (23.4)	0 (0.0)	
SD	24 (25.5)	0 (0.0)	
PD	48 (51.1)	5 (100.0)	
n/a	0 (0.0)	0 (0.0)	
Median prognostic month			n/a
irPFS	2.7 month	3.6 month	
irOS	9.5 month	9.9 month	

Table S2. Demographic features of the prospective validating cohort classified by ANO1 status.

48-case validating cohort		ANO1 status (by IHC)		
n (%)		Negative (-/+)	Positive (++/+++)	*P* value
Total		28	20	
Gender				0.537
	male	20 (71.4)	12(60.0)	
	female	8 (8.6)	8 (40.0)	
Age				0.394
	≥60	13 (46.4)	12 (60.0)	
	<60	15 (53.6)	8 (40.0)	
Tumor type				0.071
	Esophageal cancer	3 (10.7)	1 (5.0)	
	Gastric cancer	17 (60.7)	18 (90.0)	
	Colorectal cancer	8 (28.6)	1 (5.0)	
	Others	0 (0.0)	0 (0.0)	
Stage				1.0000
	I	0 (0.0)	0 (0.0)	
	II	0 (0.0)	0 (0.0)	
	III	1 (3.6)	0 (0.0)	
	IV	15 (53.6)	12 (60.0)	
	n/a	12 (42.9)	8 (40.0)	
HER2 positivity				0.744
	positive	6 (21.4)	7 (35.0)	
	negative	15 (53.6)	13 (65.0)	
	n/a	7 (25.0)	0 (0.0)	
CPS				0.680

0	5 (17.9)	4 (20.0)	
≥1	6 (21.4)	8 (40.0)	
n/a	17 (60.7)	8 (40.0)	
MSI/MMR status			0.010
MSI/dMMR	7 (25.0)	0 (0.0)	
MSS/pMMR	16 (57.1)	20 (55.6)	
n/a	5 (17.9)	0 (0.0)	
Regimen			0.765
anti-PD-1/PD-L1	9 (32.1)	4 (20.0)	
anti-PD-1/PD-L1 plus anti-CTLA4	3 (10.7)	1 (5.0)	
anti-PD-1/PD-L1 plus chemotherapy	5 (17.9)	5 (25.0)	
anti-PD-1/PD-L1 plus targeted therapy	8 (28.6)	8 (40.0)	
anti-PD-1/PD-L1 plus chemotherapy plus targeted therapy	3 (10.7)	2 (10.0)	
Therapeutic line			0.722
1	15 (53.6)	11 (55.0)	
2	5 (17.9)	2 (10.0)	
3 or higher	8 (28.6)	7 (35.0)	
Best response			0.392
CR/PR	16 (57.1)	9 (45.0)	
SD	9 (32.1)	7 (35.0)	
PD	2 (7.1)	4 (20.0)	
n/a	1 (3.6)	0 (0.0)	
Median prognostic month			n/a
irPFS	14.9 month	5.7 month	
irOS	Not reached	Not reached	

Table S3. Univariate analysis for ANO1's correlation with immunotherapeutic outcomes in the training cohort.

99-case training cohort	irPFS		irOS	
Univariate analysis	Hazard ratio	*P* value	Hazard ratio	*P* value
ANO1	2.884	0.025	2.424	0.143

Table S4. Univariate analysis for ANO1's correlation with immunotherapeutic outcomes in the validating cohort.

48-case validating cohort	irPFS		irOS	
Univariate analysis	Hazard ratio	*P* value	Hazard ratio	*P* value
ANO1	2.844	0.007	5.579	0.041
